# Neoadjuvant chemoradiation alters biomarkers of anticancer immunotherapy responses in locally advanced rectal cancer

**DOI:** 10.1136/jitc-2020-001610

**Published:** 2021-03-10

**Authors:** Incheol Seo, Hye Won Lee, Sang Jun Byun, Jee Young Park, Hyeonji Min, Sung Hwan Lee, Ju-Seog Lee, Shin Kim, Sung Uk Bae

**Affiliations:** 1 Department of Microbiology, Dongguk University College of Medicine, Gyeongju, Gyeongsangbuk-do, Korea (the Republic of); 2 Department of Pathology, Keimyung University Dongsan Medical Center, Daegu, Korea (the Republic of); 3 Institute for Cancer Research, Keimyung University, Daegu, Korea (the Republic of); 4 Department of Radiation Oncology, Keimyung University School of Medicine, Daegu, Korea (the Republic of); 5 Department of Immunology, Keimyung University School of Medicine, Daegu, Korea (the Republic of); 6 Department of Surgery, CHA University - Bundang Campus, Seongnam, Gyeonggi-do, Korea (the Republic of); 7 Department of Systems Biology, The University of Texas MD Anderson Cancer Center, Houston, Texas, USA; 8 Institute of Medical Science & Institute for Cancer Research, Keimyung University, Daegu, Korea (the Republic of); 9 Department of Surgery, Keimyung University Dongsan Medical Center, Daegu, Korea (the Republic of)

**Keywords:** radiotherapy, immunomodulation, radioimmunotherapy, gene expression profiling, genome instability

## Abstract

**Background:**

Neoadjuvant chemoradiation therapy (CRT) is a widely used preoperative treatment strategy for locally advanced rectal cancer (LARC). However, a few studies have evaluated the molecular changes caused by neoadjuvant CRT in these cancer tissues. Here, we aimed to investigate changes in immunotherapy-related immunogenic effects in response to preoperative CRT in LARC.

**Methods:**

We analyzed 60 pairs of human LARC tissues before and after irradiation from three independent LARC cohorts, including a LARC patient RNA sequencing (RNA-seq) dataset from our cohort and GSE15781 and GSE94104 datasets.

**Results:**

Gene ontology analysis showed that preoperative CRT significantly enriched the immune response in LARC tissues. Moreover, gene set enrichment analysis revealed six significantly enriched Kyoto Encyclopedia of Genes and Genomes pathways associated with downregulated genes, including mismatch repair (MMR) genes, in LARC tissues after CRT in all three cohorts. Radiation also induced apoptosis and downregulated various MMR system-related genes in three colorectal cancer cells. One patient with LARC showed a change in microsatellite instability (MSI) status after CRT, as demonstrated by the loss of MMR protein and PCR for MSI. Moreover, CRT significantly increased tumor mutational burden in LARC tissues. CIBERSORT analysis revealed that the proportions of M2 macrophages and CD8 T cells were significantly increased after CRT in both the RNA-seq dataset and GSE94104. Notably, preoperative CRT increased various immune biomarker scores, such as the interferon-γ signature, the cytolytic activity and the immune signature.

**Conclusions:**

Taken together, our findings demonstrated that neoadjuvant CRT modulated the immune-related characteristics of LARC, suggesting that neoadjuvant CRT may enhance the responsiveness of LARC to immunotherapy.

## Background

Neoadjuvant chemoradiation therapy (CRT) and total mesorectal excision are commonly incorporated into the multimodal treatment of locally advanced rectal cancer (LARC). This therapeutic strategy is associated with downstaging of the tumor, more frequent use of sphincter-preserving procedures by preoperatively shrinking the tumor, decreased local recurrence and reduced toxicity compared with postoperative adjuvant chemoradiation.^1–4^ CRT delivers ionizing radiation directly to target cells, with the goal of causing genetic damage, such as radiation-induced DNA double-strand breaks, which are repaired through double-strand break repair mechanisms.^5^ In addition to the direct cytotoxic effects of radiotherapy, this treatment may increase neoantigens, activate the major histocompatibility complex class I system, activate tumor-infiltrating lymphocytes and induce the abscopal effect, wherein localized radiation provokes distant antitumor effects.^6–9^ The abscopal effect can be enhanced by combining an immune checkpoint blocker (ICB) with radiotherapy.^10^

Cancer immunotherapy with ICBs, such as antiprogrammed death-1/programmed death-ligand 1 and anticytotoxic T lymphocyte antigen-4 inhibitors, is a novel treatment strategy in the field of immuno-oncology.^11^ To improve the effectiveness of this treatment strategy, several studies have recently evaluate various biomarkers, such as microsatellite instability (MSI),^12 13^ mismatch repair (MMR) deficiency,^13^ tumor mutational burden (TMB)^14 15^ and immune biomarker scores,^16–18^ for prediction of responses to immunotherapies, such as ICBs, in cancer.

Identifying molecular signatures using next-generation sequencing (NGS) has become an emerging focus in the field of cancer research, facilitating the diagnosis and prediction of prognosis for patients. However, a few studies have performed transcriptomic profiling of biomarkers for predicting responses to immunotherapy in rectal cancer before and after preoperative CRT. Accordingly, in this study, we investigated changes in the molecular profiles of LARC after CRT with regard to improved responses to immunotherapy through bioinformatics analysis of NGS transcriptome and Gene Expression Omnibus (GEO) datasets. Furthermore, we validated the results from these analyses using colorectal cancer (CRC) cell lines.

## Methods

### Patients and sample collection

**Figure 1:**
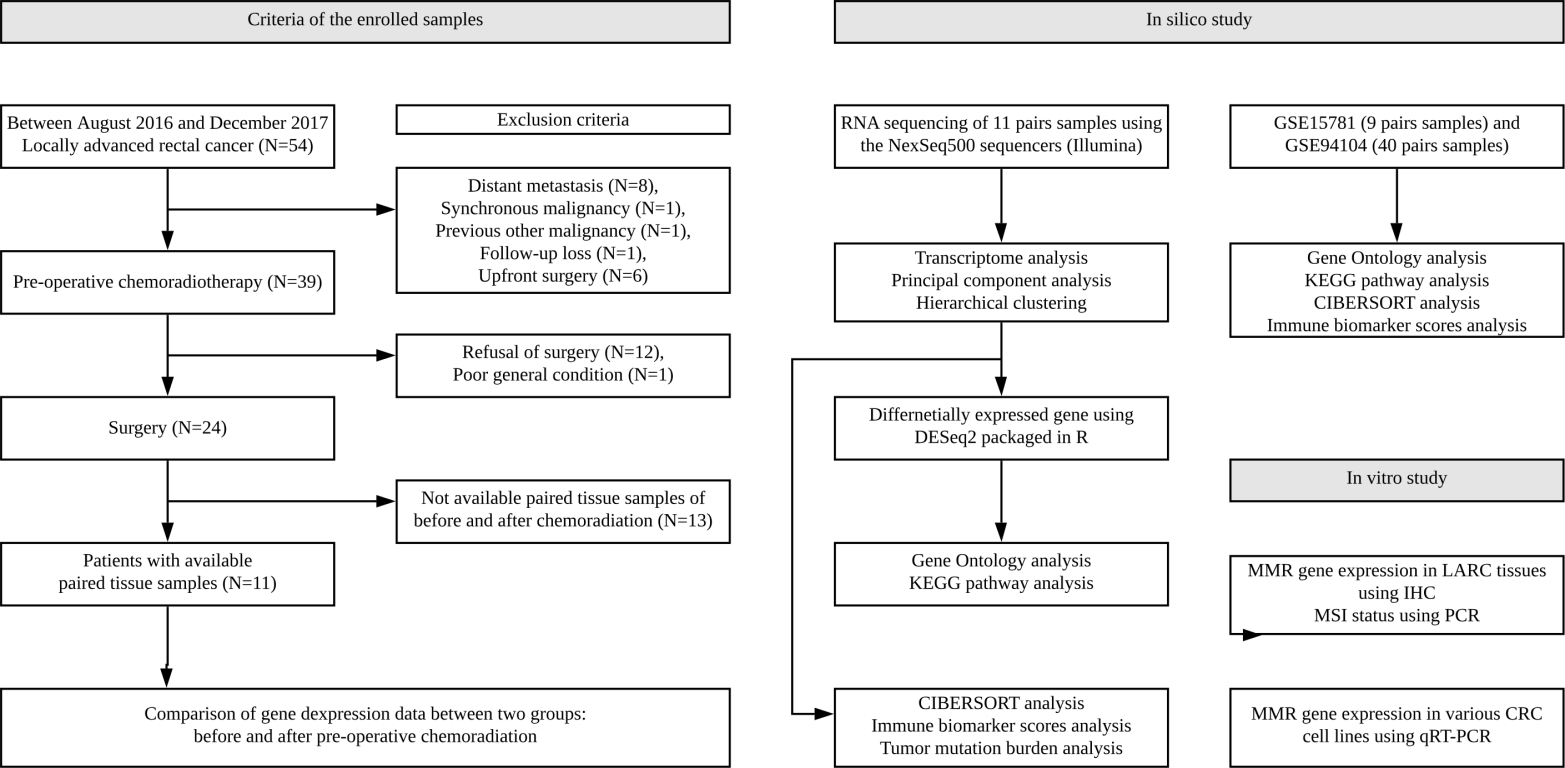
Diagram of patient selection and study workflow. Samples before neoadjuvant CRT were obtained at the time of diagnosis using endoscopy. Samples after neoadjuvant CRT were obtained after surgical resection. Samples were obtained from 11 patients after enrollment and were prepared and used for RNA sequencing analysis. The 11 patients underwent curative surgery. CRC, colorectal cancer; CRT, chemoradiation therapy; IHC, immunohistochemistry; KEGG, Kyoto Encyclopedia of Genes and Genomes; LARC, locally advanced rectal cancer; MMR, mismatch repair; MSI, microsatellite instability.

Formalin-fixed paraffin-embedded (FFPE) block specimens from preoperative biopsy via sigmoidoscopy and surgical resection of the primary tumor were obtained from 54 patients with rectal adenocarcinoma who underwent neoadjuvant concurrent CRT between August 2016 and December 2017. Preoperative clinical stage was determined by abdominal and chest CT scans and pelvic MRI and the inclusion criterion was clinical stages 2–3 (T3 or T4 and/or node positive) rectal adenocarcinoma. The exclusion criteria were stage IV tumors, cancer related to familial adenomatous polyposis or hereditary nonpolyposis CRC, synchronous or previous malignancies, distant metastasis during neoadjuvant CRT and patients who refused the definite surgery or were lost to follow-up. Based on these criteria, we included 11 pairs of human CRC tissues obtained before and after CRT (online supplemental table 1) and performed RNA sequencing (RNA-seq) analysis. The detailed study design and enrolled samples at each stage are illustrated in the work flow shown in figure 1. All patients received conventional long-course neoadjuvant CRT (five cycles of 5-fluorouracil (5-FU)-based chemotherapy and 50.4 Gy radiation) online supplemental method 1. Additionally, all patients underwent staging abdominopelvic and chest CT scan, rectal MRI, colonoscopy, biopsy and positron emission tomography scans. Total mesorectal excision was performed within 6–8 weeks of the final CRT.

10.1136/jitc-2020-001610.supp1 Supplementary data

10.1136/jitc-2020-001610.supp8 Supplementary data

### Cell cultures and materials

DLD-1 and SW480 human colorectal adenocarcinoma cells and HCT-116 human CRC cells were obtained from the American Type Culture Collection (Rockville, Maryland, USA) and grown in GIBCO RPMI1640 medium (Thermo Fisher Scientific, Wilmington, Delaware, USA) supplemented with 10% heat-inactivated fetal bovine serum (Thermo Fisher Scientific), 2 mM l-glutamine, 100 µg/mL streptomycin and 100 µg/mL penicillin. 5-FU was purchased from Sigma-Aldrich (St. Louis, Missouri, USA).

### Gene expression databases

The rectal cancer gene expression profiles used in this study were downloaded from the publicly available GEO database (National Institutes of Health, Bethesda, Maryland, USA; http://www.ncbi.nlm.nih.gov/geo/), with accession numbers GSE15781^19^ and GSE94104.^20^ The gene expression profiles (ID, GSE15781) were previously produced using the ABI Human Genome Survey Microarray V.2 (Thermo Fisher Scientific). Briefly, specimens from GSE15781 were obtained from 13 patients with LARC who received 50 Gy (delivered as 25 fractions of 2 Gy) over a 5-week period. In addition, patients received Capecitabine (Xeloda; Roche) 2500 mg/mL daily throughout the treatment period. Resection of the rectum was performed 4–6 weeks after preoperative radiation therapy. Among the specimens in the GSE15781 dataset, nine paired tumor tissues before and after CRT were analyzed in this study. An additional gene expression profile (ID, GSE94104) was previously produced using an Illumina Human HT-12 WG-DASL V4.0 R2 expression beadchip (Illumina, San Diego, California, USA). Briefly, the specimens in the GSE94104 dataset were obtained from 40 patients with LARC who received 45 Gy in 25 fractions over a 5-week period along with 5-FU or capecitabine. Patients underwent total mesorectal excision at an interval of 1–14 weeks (mean=9 weeks) following treatment. Prior to bioinformatics analysis, we converted the probe ID to HUGO gene symbols (accessed on December 15 2019) according to the annotation information for the probes from platforms GPL2986 and GPL14951. When there were multiple probe IDs for one gene, only the probe ID with the largest absolute deviation between samples was selected. The probe intensity was normalized using the quantile normalization method and then log_2_-transformed for gene expression analysis. For gene set enrichment analysis (GSEA) and CIBERSORT analyses, natural scale probe intensities were used.

### RNA extraction, library preparation and sequencing

Total RNA was extracted using TRIzol RNA Isolation Reagent (Life Technologies) from the 11 pairs of human LARC. The quantity and quality of the total RNA were evaluated using an Agilent 2100 bioanalyzer RNA kit (Agilent). The isolated total RNA was processed for preparation of an RNA-seq library using an Illumina TruSeq Stranded mRNA Sample Preparation kit (Illumina) according to the manufacturer’s protocol. The quality and size of libraries were assessed using an Agilent 2100 bioanalyzer DNA kit (Agilent). All libraries were quantified by quantitative real-time PCR (qPCR) using a CFX96 Real Time System (Bio-Rad Laboratories, Hercules, California, USA) and sequenced on NextSeq500 sequencers (Illumina) with a paired-end 76 bp plus single 6 bp index read run.

### RNA-seq, quality control and mapping

For quality control, low-quality bases and adapter sequences were trimmed from the raw sequencing reads using Trimmomatic software^21^ with default parameters, except that the minimum length of reads to be dropped was 38 bp (online supplemental table 2). Trimmed sequencing reads were mapped to the hg38 human reference genome^22^ using STAR aligner^23^ with default parameters. Reads per gene was counted simultaneously by STAR using the ‘--quantMode GeneCounts’ parameter.

10.1136/jitc-2020-001610.supp2 Supplementary data

### Analysis of differentially expressed genes

Count normalization and differentially expressed gene (DEG) identification in pairwise comparisons between before and after CRT were performed using R software V.3.5.0 (The R Foundation for Statistical Computing, 2018) using DESeq2 package.^24^ For GSE15781 and GSE94104, we used limma package^25^ with default parameters. DEGs were defined as satisfying both fold change greater than or equal to two and Benjamini and Hochberg adjusted p value less than 0.05.

### Gene ontology and Kyoto Encyclopedia of Genes and Genomes pathway analyses

The biological significance of DEGs was analyzed using The Database for Annotation, Visualization, and Integrated Discovery (DAVID) 6.8 (http://david.ncifcfr.gov, accessed on May 15 2020).^26^ The bar plot was generated to show significantly enriched terms of designated genes involved in biological processes. GSEA was performed^27^ for 186 curated Kyoto Encyclopedia of Genes and Genomes (KEGG) gene sets (MSigDB collection name: CP:KEGG of C2) with default parameters, except that the permutation type was set to ‘gene_set’. The significance cut-off for the false-discovery rate q value was set to 0.05.

### Analysis of tumor-infiltrating immune cells

The relative proportions of the 22 tumor-infiltrating immune cells (TIICs) in CRC tissues were estimated using CIBERSORT (http://cibersort.stanford.edu/)^28^ with the leucocyte gene signature matrix (LM22). CIBERSORT analysis was carried out with DESeq2-normalized counts for RNA-seq and natural scale probe intensity for arrays using 500 permutations with quantile normalization. The abundance weighted Bray-Curtis dissimilarity was calculated from the relative proportions of TIICs in each sample. Then, a permutation multivariate analysis of variance with 1000 permutations was conducted to test statistical differences in TIIC compositions between before and after CRT. Principal component analysis (PCA) was performed on the ratio of TIICs in order to explore the similarities among samples. A volcano plot showing the fold change ratios compared with Wilcoxon signed-rank test’s p values was created to identify statistically different TIICs.

### Analysis of immune biomarker scores

Three immune biomarker scores were calculated to compare the immune activities of the specimens before and after neoadjuvant CRT. The average of log_2_-transformed normalized expression counts or intensities (geometric mean) of the genes listed below was used for each score calculation. The gene lists included in each score calculation were as follows: *CXCL9*, *CXCL10*, *IDO1*, *IFNG*, *HLA-DRA*, and *STAT1; GZMA* and *PRF1*; and *CD247*, *CD2*, *CD3E*, *GZMH*, *NKG7*, *PRF1* and *GZMK* for the interferon-γ (IFN-γ) signature,^16^ the cytolytic activity^17^ and the immune signature,^18^ respectively.

### TMB estimation

To evaluate changes in TMB before and after neoadjuvant CRT using RNA-seq data, the following three steps were performed. First, variants, including single-nucleotide polymorphisms (SNPs) and small insertions and deletions, were called from the RNA-seq data according to GATK’s best practice (https://gatk.broadinstitute.org/hc/en-us/articles/360035531192-RNAseq-short-variant-discovery-SNPs-Indels). Briefly, sequencing reads were mapped to hg38 using STAR aligner two-pass mode with the ‘sjdbOverhang=75’ parameter. The Picard tool (http://broadinstitute.github.io/picard/) was used for adding read group information, sorting, marking duplicates and indexing to the mapped reads. Mapped reads were split into exon segments, and any overhanging reads were hard-clipped into the intronic regions using SplitNCigarReads tool. Base quality scores were recalibrated based on build 138 of the dbSNP using BaseRecalibrator tool. Variant calling and filtering were performed using HaplotypeCaller and VariantFiltration tools, respectively. The number of variants called from each sample is described in online supplemental table 3. Second, to exclude germline mutations from the called variants, shared mutations between samples collected before and after CRT for a single patient were listed and filtered using the CombineVariants tool in the GATK toolkit. Finally, the number of somatic variants was normalized to the total number of reliable bases having sufficient mapped transcripts of respective sequencing samples because the number of somatic variants estimated from RNA-seq data could not be normalized by the size of the whole exome as in the conventional method.^29^ The total number of reliable bases for each sample was counted using the CallableLoci tool in the GATK toolkit. Finally, normalized values were converted into mutations per million base pairs, a conventional unit for the TMB.

10.1136/jitc-2020-001610.supp3 Supplementary data

### Chemoradiation

To estimate the response to chemoradiation, DLD-1 (0.1×10^6^ cells/well), HCT-116 (0.07×10^6^ cells/well) and SW480 (0.15×10^6^ cells/well) cells were plated in 6-wells plates and incubated at 37°C under humidified conditions in an atmosphere containing 5% CO_2_. The cells were treated with 2.5 µM 5-FU and/or without irradiated with 10 or 20 Gy X-rays radiation in one or two fractions (6 mega-voltage; dose rate: 200 cGy/s) using a linear accelerator (VitalBeam; Varian, USA) for two consecutive days. After radiation, culture medium was exchanged to exclude the effects of radiation on the contents of the culture medium.

### RNA isolation and reverse transcription-qPCR

Total cellular RNA was extracted from tissues using TRIzol reagent (Molecular Research Center, Inc., Cincinnati, Ohio, USA). RNA was quantified using a NanoDrop 1000 (Thermo Fisher Scientific). cDNA was synthesized from 2 µg total RNA using MMLV reverse transcriptase (Promega, Madison, Wisconsin, USA) according to the manufacturer’s protocol. qPCR was performed on a LightCycler 480 real-time PCR system (Roche Diagnostics, Mannheim, Germany) using the specific primer pairs described in table 1 and SYBR Green Premix (Toyobo, Japan). β-Actin and β-glucuronidase were used as housekeeping genes for normalization, and a no-template sample was used as a negative control. qPCR data were analyzed using ∆Ct values.^30^ All experiments were performed with three replicates, and similar results were obtained.

**Table 1:** Primer sequences of mismatch repair-related genes evaluated by quantitative PCR

Primer name	Sequences
EXO1 sense	5’-TGAGGAAGTATAAAGGGCAGGT −3’
EXO1 antisense	5’-AGTTTTTCAGCACAAGCAATAGC-3’
MLH1 sense	5’-CTCTTCATCAACCATCGTCTGG-3’
MLH1 antisense	5’-GCAAATAGGCTGCATACACTGTT −3’
LIG1 sense	5’-GAAGGAGGCATCCAATAGCAG −3’
LIG1 antisense	5’-ACTCTCGGACACCACTCCATT-3’
MLH3 sense	5’-ACAAGCCAAATTGCGTTCTGG −3’
MLH3 antisense	5’-TTCAGCATCAATACTGTTGAGGG −3’
MSH2 sense	5’-AGGCATCCAAGGAGAATGATTG −3’
MSH2 antisense	5’-GGAATCCACATACCCAACTCCAA −3’
MSH3 sense	5’-GTGGACCCCGGATATAAGGTGGG −3’
MSH3 antisense	5’-AAAGGGCAGTCAATTTCCGGG −3’
MSH6 sense	5’-CCAAGGCGAAGAACCTCAAC −3’
MSH6 antisense	5’-ACCAGGGGTAACCCTCCATC −3’
PCNA sense	5’-CCTGCTGGGATATTAGCTCCA −3’
PCNA antisense	5’-CAGCGGTAGGTGTCGAAGC −3’
PMS2 sense	5’-CCTATTGATCGGAAGTCAGTCCA −3’
PMS2 antisense	5’-CTACTAACTCCTTTACCGCAGTG −3’
POLD1 sense	5’-ATCCAGAACTTCGACCTTCCG −3’
POLD1 antisense	5’-ACGGCATTGAGCGTGTAGG −3’
POLD2 sense	5’-CCATCAGCCAACAATGCCAC −3’
POLD2 antisense	5’-CTAGCCGGAAGGGTTGTGA −3’
POLD3 sense	5’-GAGTTCGTCACGGACCAAAAC −3’
POLD3 antisense	5’-GCCAGACACCAAGTAGGTAAC −3’
POLD4 sense	5’-ACCCAAGAACCTCAGGACAG −3’
POLD4 antisense	5’-AGTTGAGCCTCTGACACCTC −3’
RFC1 sense	5’-TGGAGAGGCAGTTGCATGAAG −3’
RFC1 antisense	5’-CCTTTCGAGCCTTTTTGGTCT −3’
RFC2 sense	5’-GTGAGCAGGCTAGAGGTCTTT −3’
RFC2 antisense	5’-TGAGTTCCAACATGGCATCTTTG −3’
RFC3 sense	5’-GTGGACAAGTATCGGCCCTG −3’
RFC3 antisense	5’-TGATGGTCCGTACACTAACAGAT −3’
RFC4 sense	5’-CCGCTGACCAAGGATCGAG −3’
RFC4 antisense	5’-AGGGAACGGGTTTGGCTTTC −3’
RFC5 sense	5’-GAAGCAGACGCCATGACTCAG −3’
RFC5 antisense	5’-GACCGAACCGAAACCTCGT −3’
RPA1 sense	5’-GGGGATACAAACATAAAGCCCA −3’
RPA1 antisense	5’-CGATAACGCGGCGGACTATT −3’
RPA2 sense	5’-GCACCTTCTCAAGCCGAAAAG −3’
RPA2 antisense	5’-CCCCACAATAGTGACCTGTGAAA −3’
RPA3 sense	5’-AGCTCAATTCATCGACAAGCC −3’
RPA3 antisense	5’-TCTTCATCAAGGGGTTCCATCA −3’
RPA4 sense	5’-GTGACCAACTGTGTGAGAGAG −3’
RPA4 antisense	5’-TACACGGTACAACGTCCTGAA −3’
SSBP1 sense	5’-TGAGTCCGAAACAACTACCAGT −3’
SSBP1 sense	5’-CCTGATCGCCACATCTCATTAG −3’
β-actin sense	5’-CAGCCATGTACGTTGCTATCCAGG-3’
β-actin antisense	5’-AGGTCCAGACGCAGGATGGCATG-3’
β-glucuronidase sense	5’-CCCACTCAGTAGCCAAGTCA −3’
β-glucuronidase antisense	5’-CACAAAACCCAGGCCAGAAA −3’

### Immunohistochemistry

Immunohistochemistry (IHC) was performed for four MMR proteins (MutL homolog (MLH) 1, MSH2, MSH6 and PMS1 homolog 2 (PMS2)). All human primary LARC samples were FFPE tissues. The representative blocks were selected for each case after review of hematoxylin and eosin slides. We built sets of tissue microarrays from biopsy and paired surgical specimens. The staining was performed using a BenchMark ULTRA automated staining system (Ventana Medical Systems, Tucson, Arizona, USA), according to the manufacturer’s protocol. The monoclonal antibodies used in this study were as follows: anti-MLH1 (clone M1, ready to use), anti-MSH2 (clone G219-1129, ready to use), anti-MSH6 (clone SP93, ready to use) and anti-PMS2 (clone A16-4, ready to use); all of these antibodies were from Ventana (Roche/Ventana Medical Systems). Bound antibodies were visualized using an OptiView DAB Detection Kit (Ventana Medical Systems). All sections were evaluated by an experienced pathologist (HWL) who was blinded to the clinicopathological features or clinical outcome. Unequivocal nuclear staining of tumor cells compared with adjacent stromal cells or intratumoral lymphocytes (internal control) was regarded as positive. The absolute absence of nuclear staining was considered to indicate a defect in an MMR protein. There were no equivocal findings of IHC in the biopsy specimens. Five surgical specimens showed ambiguous findings, such as reduced expression in both tumor cells and the internal control or aberrant expression. These results could be explained by poor fixation or treatment effects.^31^ In these cases, MSI analysis using PCR was also performed for validation.

### MSI analysis

MSI assays were performed on DNA extracted from FFPE and matched normal tissues. Tumor and adjacent normal areas were separately marked and collected. Genomic DNA was extracted using a QIAamp DNA Mini Kit (Qiagen, Hilden, Germany) according to the manufacturer’s protocol. The extracted DNA was amplified by PCR with fluorescent dye-labeled primers targeting five microsatellite loci: BAT25, BAT26, D5S346, D2S123 and D17S250, as recommended by the National Cancer Institute (NCI) guidelines. For MSI analysis, differences in amplified PCR fragments between tumor and normal tissues were detected using a Qsep100 gene analyzer (Bioptic, Taiwan, China). In accordance with the NCI criteria, MSI-high tumors were defined as having instability in two or more microsatellite loci; MSI-low tumors were defined as having instability in only one locus; and microsatellite stable (MSS) tumors were defined as showing no apparent instability.

### Statistical analysis

Most statistical analyses were performed in R. Graphs, related to R statistical analyses, were drawn using the ggplot2 package^32^ in R. Differences in CRC tissues from patients with LARC before and after CRT were statistically analyzed using paired t-tests or Wilcoxon signed-rank tests. Results with p values of less than 0.05 were considered statistically significant.

## Results

### Neoadjuvant CRT induced MMR deficiency in LARC

**Figure 2:**
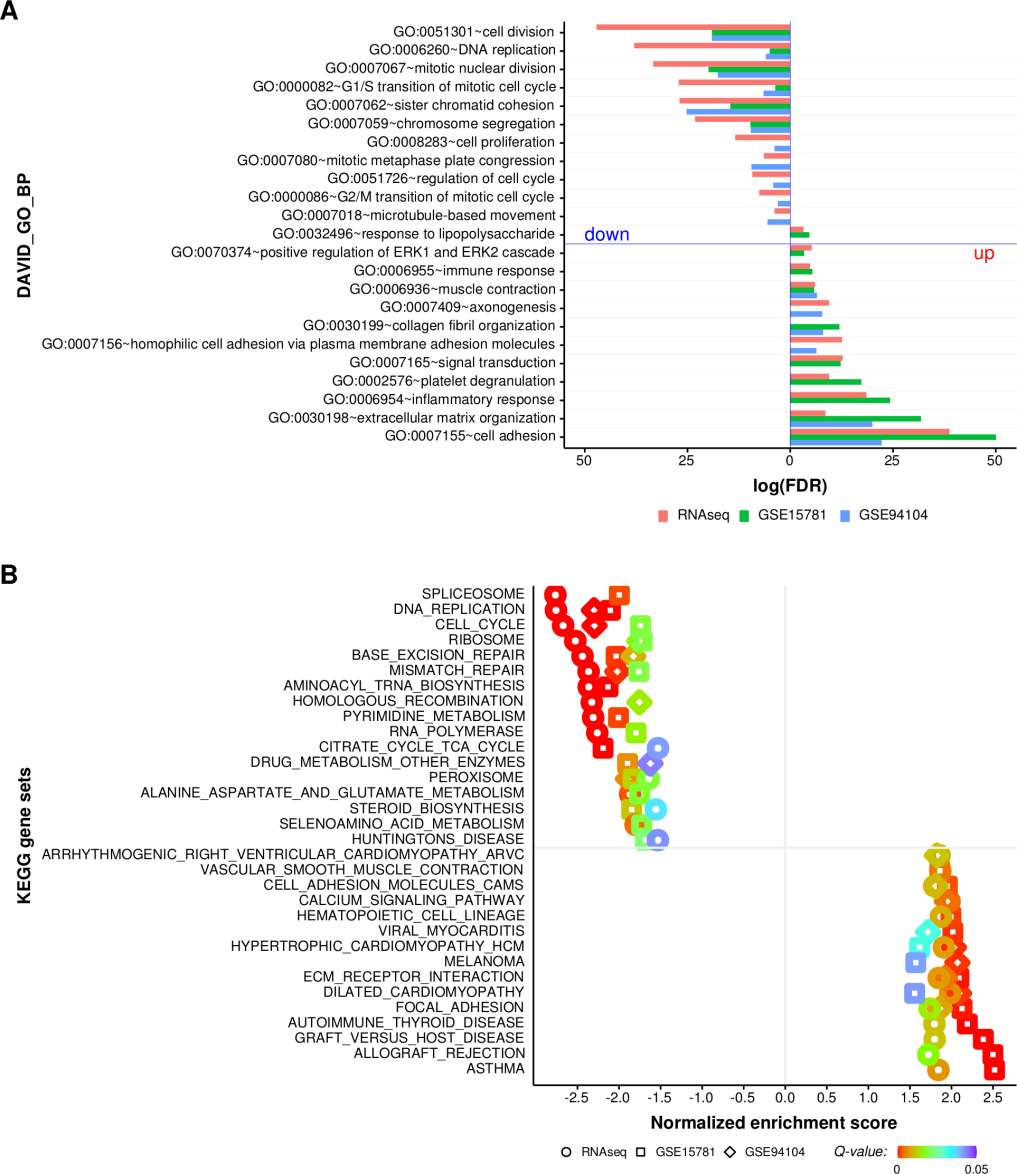
Functional enrichment analysis of differentially expressed genes (DEGs) in LARC tissues before and after CRT. (A) Functional classification of genes dysregulated in LARC tissues after CRT compared with that of LARC tissues before CRT using gene ontology classifications. The bars extending to the left and right reflect significantly downegulated and upregulated biological processes in LARC tissues after CRT, respectively. (B) Significantly enriched KEGG terms for DEGs from LARC tissues after CRT compared with that from LARC tissues before CRT. The panel shows the results of all three cohorts from LARC tissues before and after CRT using KEGG enrichment. Biological processes were ranked on the basis of normalized enrichment scores; positive and negative normalized enrichment scores indicate downregulation or upregulation, respectively, in LARC tissues after CRT. The significance cut-off for the FDR Q-value was set to 0.05. CRT, chemoradiation therapy; FDR, false-discovery rate; GO, gene ontology; KEGG, Kyoto Encyclopedia of Genes and Genomes; LARC, locally advanced rectal cancer.

To categorize biological processes of DEGs between LARC tissues before and after CRT from the same patient group, we used DAVID and three LARC datasets. As shown in figure 2A and online supplemental figure 2A, significantly enriched biological processes in the same patients with LARC after CRT compared with that before CRT were cell adhesion, extracellular matrix organization, inflammatory response, immune response and response to lipopolysaccharide. Additionally, the significantly enriched biological processes in the same patients with LARC before CRT compared with that after CRT were cell division, DNA replication, mitotic nuclear division, G_1_/S transition and sister chromatic cohesion (figure 2A and online supplemental figure 2B). To predict the functions and expression trends for genes involved in modulating the effects of CRT in LARC tissues, we applied GSEA using the three LARC cohorts. GSEA yielded a variety of gene pathways and categories that were significantly modified on average across LARC tissues after CRT. Dysregulated mRNAs were associated with six biological pathways, including DNA replication, cell cycle, ribosome, base excision repair, MMR and peroxisome, in all three cohorts (figure 2B and online supplemental figure 2C). As shown in online supplemental table 4, GSEA of RNA-seq, GSE15781 and GSE94104 datasets showed statistically significant associations with 46, 60 and 23 biological pathways, respectively. GSEA results for all three datasets showed downregulation of the MMR system, with normalized enrichment scores of −2.37 to –1.76 and −2.02, respectively (q<0.001, 0.019 and 0.001, respectively). Then, to investigate whether the 23 MMR system-related genes^27^ were altered in our cohort of LARC tissues after CRT compared with that before CRT, statistical comparisons were performed using Wilcoxon signed-rank tests for paired LARC tissues. As shown in online supplemental figure 1, the average mRNA expression levels of the 23 MMR-related genes tended to decrease in LARC tissues after CRT.

10.1136/jitc-2020-001610.supp4 Supplementary data

10.1136/jitc-2020-001610.supp5 Supplementary data

### Radiation downregulated the mRNA levels of MMR genes in various CRC cells

**Figure 3:**
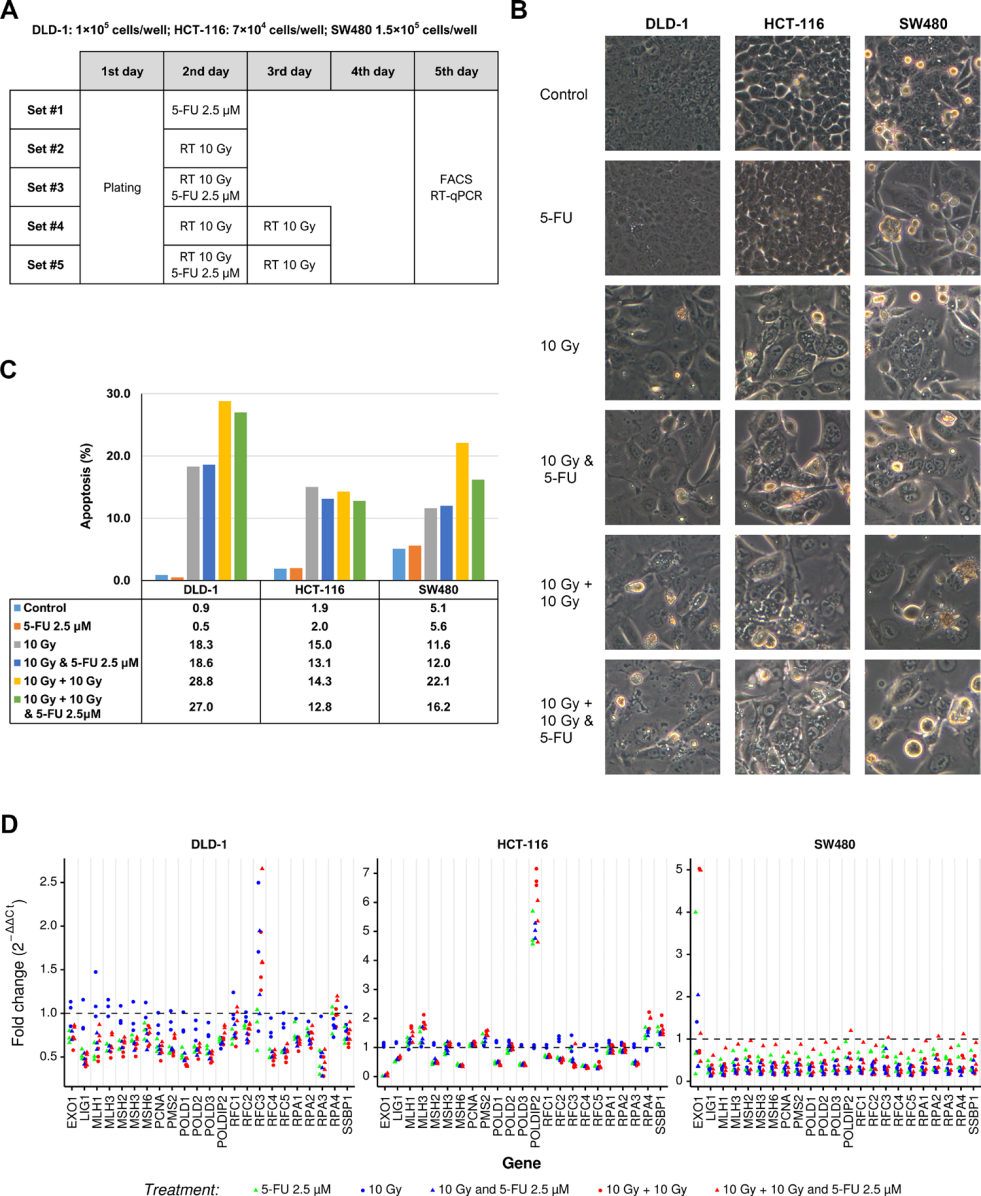
Dysregulation of 23 MMR system-related genes in CRC cell lines before and after CRT. (A) Schedule for radiation exposure. (B) Morphological changes were visualized using light microscopy (×200 magnification). (C) The sub-G_1_ fraction (apoptotic cells) was measured by flow cytometry. (D) Relative mRNA expression levels of 23 MMR system-related genes in CRC cells. 5-FU, 5-fluorouracil; CRC, colorectal cancer; CRT, chemoradiation therapy; MMR, mismatch repair; RT-qPCR, real-time quantitative PCR,

To validate the observed results regarding the effects of CRT on LARC tissues, we exposed CRC cell lines to 2.5 µM 5-FU and/or radiation (figure 3A) and then examined changes in the expression levels of MMR system-related genes in CRC cells using qPCR. Prior to investigating the effects of radiation on MMR system-related genes, we explored whether radiation induced apoptosis in various CRC cell lines, including DLD-1, HCT-116 and SW480 cells. As shown in figure 3B, C, radiation induced morphological changes consistent with apoptosis, such as shrinkage, cellular detachment from the plate and rounding of cells, and increased accumulation of sub-G_1_-phase cells. Importantly, as demonstrated in figure 3D, mRNA expression levels of almost the 23 MMR system-related genes were markedly decreased, whereas the mRNA levels of *EXO1*, *MLH1* and *MLH3* at 10 Gy and *RFC3* were not decreased in DLD-1 cells. Moreover, the mRNA expression levels of *EXO1*, *LIG1*, *MSH2*, *MSH6*, *POLD1*, *POLD3*, *RFC1*, *RFC2*, *RFC3*, *RFC4*, *RFC5* and *RPA3* mRNAs were markedly decreased in HCT-116 cell lines treated with 5-FU, 10 Gy and 5-FU, and 10 Gy, 10 Gy, and 5-FU. Additionally, in SW480 cells treated with 5-FU and/or radiation, a remarkable decrease was observed in all genes except *EXO-1*.

### Neoadjuvant CRT altered MSI status and increased TMB in LARC tissues

**Figure 4:**
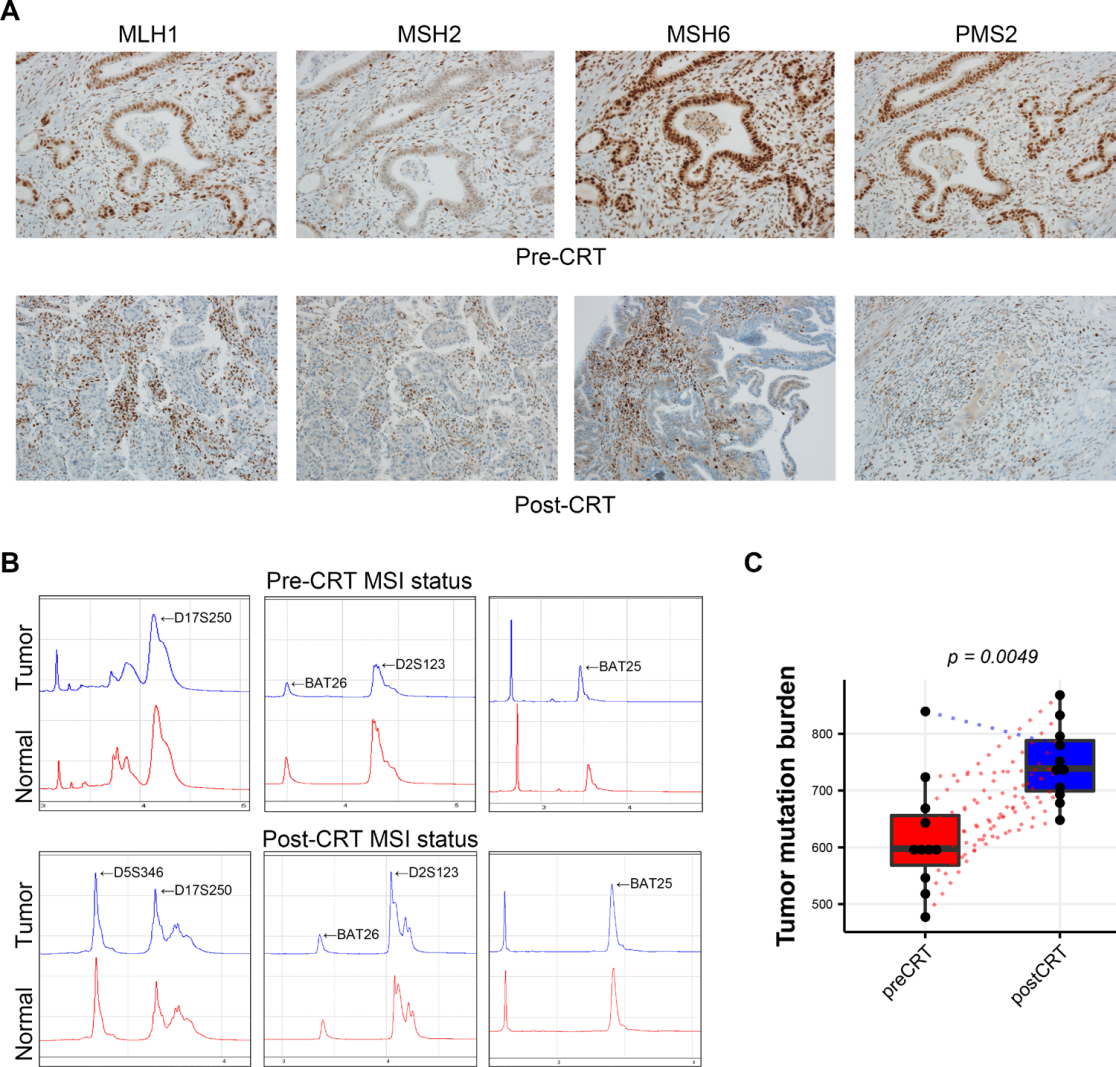
Comparison of MSI status and TMB in LARC tissues before and after CRT. (A) Immunohistochemistry for MMR proteins (×200 magnification). (B) DNA electropherograms of MSI status. (C) Comparison of TMB between LARC tissues before and after CRT. CRT, chemoradiation therapy; LARC, locally advanced rectal cancer; MSI, microsatellite instability; TMB, tumor mutational burden.

To investigate the effects of CRT on MSI status and TMB in LARC tissues, biopsy specimens of LARC tissues before CRT were evaluated. As shown in figure 4A, nuclear expression was maintained in tumor cells for all four MMR system-related proteins, including MLH1, MSH2, MSH6 and PMS2. Moreover, neoadjuvant CRT resulted in loss of MSH6 in one of 11 LARC tissues (figure 4A). In this same case, DNA electropherograms showed that MSI-low was detected in LARC tissue after CRT, whereas the LARC specimen before CRT for the same patient showed MSS (figure 4B). Furthermore, the TMB was significantly higher in LARC tissues after CRT (p=0.0049) than in that before CRT (figure 4C).

### Comparative analysis of TIIC compositions in LARC tissues before and after CRT

**Figure 5:**
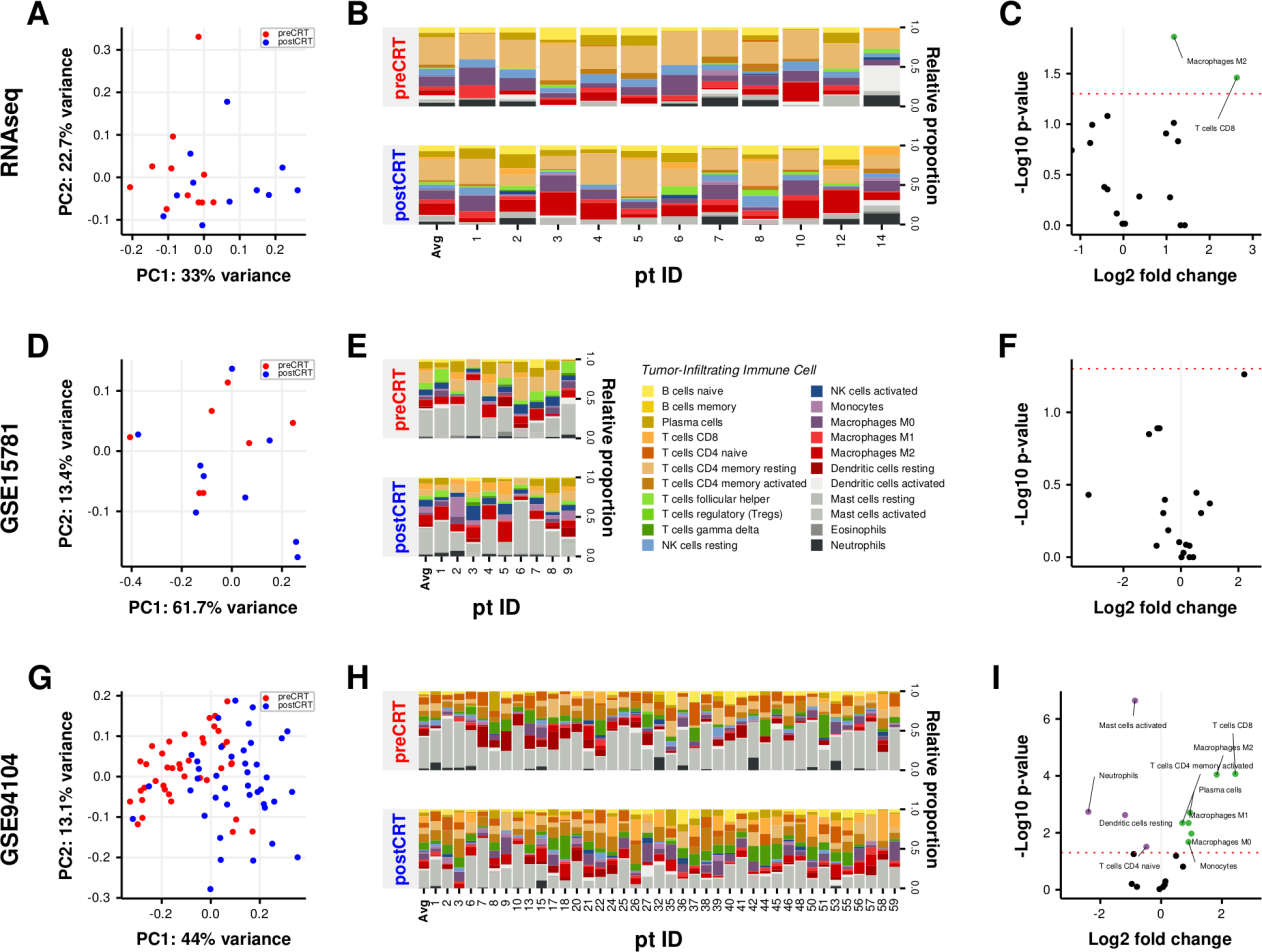
Comparison of tumor-infiltrating immune cell fractions in LARC tissues before and after CRT. (A) PCA of TIIC fractions from each sample before and after CRT. The first two principal components explaining most of the data variation are shown. (B) Differences in TIICs between paired LARC tissues before and after CRT. (C) Volcano plot visualizing the differential TIICs. The green and purple points in the plot represent subpopulations with significant differences (p<0.05). CRT, chemoradiation therapy; LARC, locally advanced rectal cancer; PCA, principal component analysis; RNAseq, RNA sequencing; TIICs, tumor-infiltrating immune cells.

To determine the diversity and landscape of TIICs, a gene expression-based deconvolution algorithm, CIBERSORT, was applied to the three cohorts. First, we performed PCA on the relative proportions of TIICs to visualize the effects of irradiation. As shown in figure 5A, D and G, PCA plots exhibited similar compositional shifts in TIICs in all three cohorts. Next, to investigate which TIIC fractions were significantly changed by CRT, we analyzed the proportions of TIICs in the three cohorts. As shown in figure 5C and I, we found that the proportions of M2 macrophages (p=0.01367 and p=0.00009) and CD8 T cells (p=0.03461 and p=0.00009) were significantly increased after CRT in both the RNA-seq and GSE94104 datasets, respectively. Moreover, the proportions of activated memory CD4 T cells, plasma cells, M0 and M1 macrophages, and monocytes in the GSE94104 dataset (p=0.00451, p=0.00196, p=0.01070, p=0.00454 and p=0.02100, respectively, figure 5I) were also significantly altered. Analysis of the GSE94104 dataset showed that the proportions of activated mast cells, neutrophils, resting dendritic cells and naïve CD4 T cells were significantly decreased after CRT in the GSE94104 dataset (p<0.00001, p=0.00183, p=0.00238 and p=0.03098, respectively; figure 5I).

### Analysis of immune biomarker scores in LARC tissues before and after CRT

**Figure 6:**
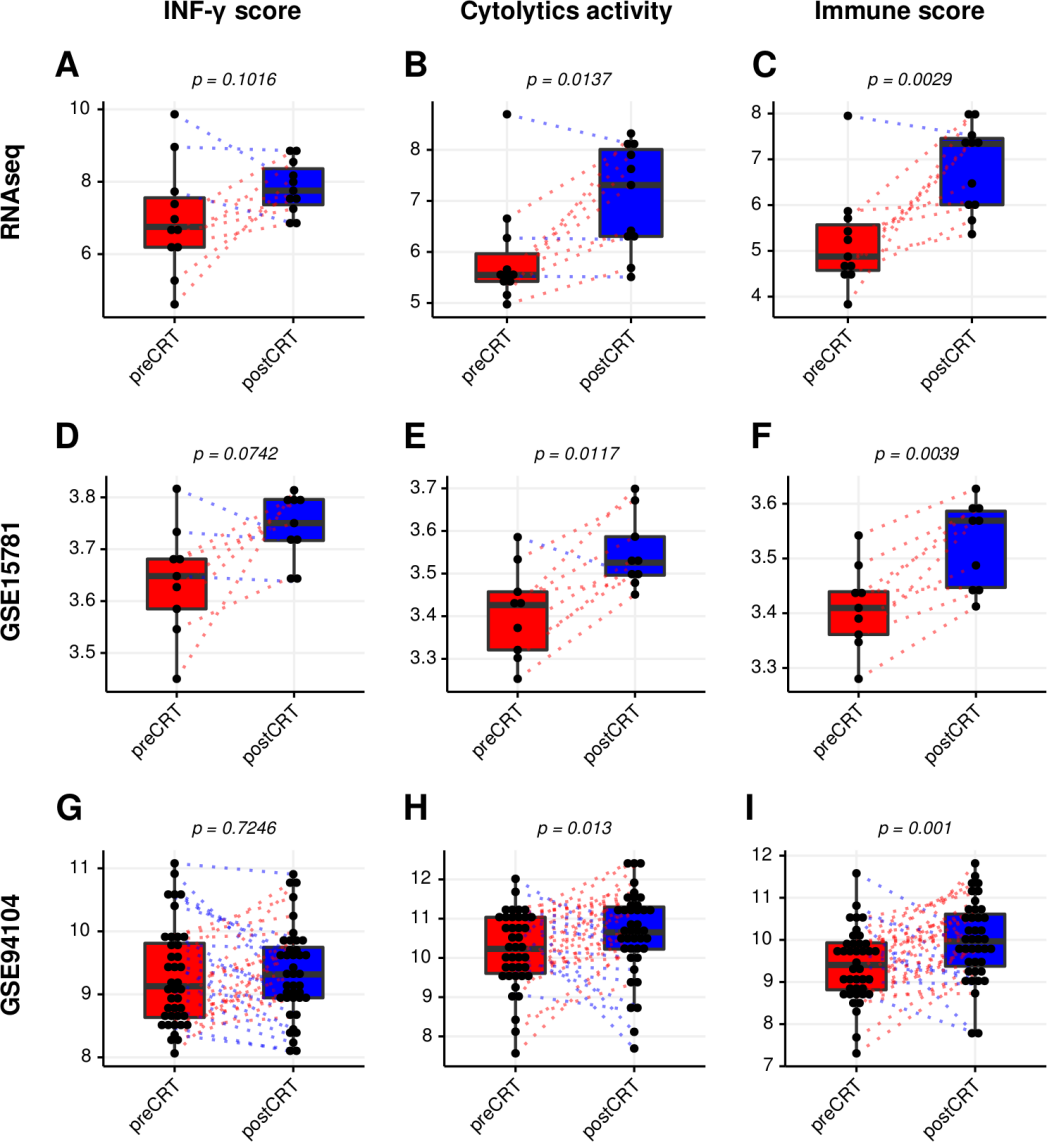
Comparison of immune biomarker scores in LARC before and after CRT. Immune scores of the interferon (IFN)-γ signature. (A) The cytolytic activity (D) and the immune signature (G) from the RNAsequencing cohort. Immune scores of the IFN-γ signature (B), the cytolytic activity (E), and the immune signature (H) from the GSE15781 dataset. immune scores of the IFN-γ signature (C), the cytolytic activity (F), and the immune signature (I) from the GSE94104 dataset. CRT, chemoradiation therapy; LARC, locally advanced rectal cancer; RNAseq, RNA sequencing.

Accumulating evidence has demonstrated that immune biomarker scores could be used to predict responses to immunotherapy.^16–18^ To investigate the possibility of improving responsiveness to immunotherapy after CRT, three immune biomarker scores (the IFN-γ signature, the cytolytic activity and the immune signature) before and after CRT were analyzed. Scores for the cytolytic activity and the immune signature were significantly increased in all three datasets (figure 6B, C, E, F, H, and I), and the IFN-γ signature tended to increase after CRT in the RNA-seq and GSE15781 dataset (figure 6A–D).

## Discussion

Neoadjuvant CRT is a promising therapeutic strategy for patients with LARC to increase rates of tumor downstaging, clinical response and pathological response and to improve surgical resectability. The main mechanism mediating the anticancer effects of radiotherapy is direct tumor cell killing owing to double-strand breakage of DNA in cancer cells.^33^ Nevertheless, recent studies have reported that radiotherapy also has anticancer effects by activation of the immune system through regulation of tumor immunogenicity,^9 34^ including improving intratumoral immune cell infiltration,^35^ enriching neoantigens,^36^ generating tumor-associated antigen-specific immune cells,^37^ facilitating the activity of major histocompatibility complex class I by increasing the intracellular peptide pool^35^ and releasing high-mobility group protein box 1.^38^ ICBs were approved by the US Food and Drug Administration as innovative anticancer therapies,^39^ and synergistic therapeutic strategies using ICBs and radiotherapy have been explored for the treatment of various types of cancer,^36^ including hepatocellular carcinoma,^40^ CRC,^41^ breast cancer,^41^ oral cancer^42^ and esophageal cancer.^43^ As the importance of precision medicine has become clear, studies have also evaluated the effects of CRT on various bioinformatic biomarkers that can predict the reactivity of human cancers to ICBs. Accordingly, we designed this study using three cohorts to explore whether CRT could alter the expression levels of potential biomarkers of the response to immunotherapies, such as ICBs, in LARC.

In this study, we investigated the immune-related efficacy of CRT in LARC and evaluated whether CRT increased immunogenicity or induced immune system activation. After processing our transcriptomic data, we first performed gene ontology (GO) analysis and functional enrichment analysis in our LARC cohort. The results revealed that preoperative CRT significantly enriched the immune response (GO:0006955) in LARC tissues. Additionally, from our CIBERSORT analyses, we found that the proportions of CD8+ T cells and M2 macrophages were significantly increased in the LARC microenvironment after CRT. Ionizing radiation has been shown to recruit M2 macrophages to radiated tissues through directly increasing epithelial-to-mesenchymal transition-related markers and secretion of various chemokines.^44^ Therefore, it is possible that CRT may induce the recruitment of M2 macrophages into the LARC microenvironment. However, further studies are needed to investigate the signaling mechanisms and roles of M2 macrophages in the LARC microenvironment after CRT.

Among GO classifications, biological adhesion involves the adhesion of the symbiont to the host, cell adhesion, intermicrovillar adhesion and multicellular organism adhesion.^45^ Additionally, radiotherapy leads to upregulation of intercellular adhesion molecule 1 and vascular cell adhesion molecule 1 in the tumor microenvironment.^46^ Our results demonstrated that CRT enriched the biological adhesion process and enhanced focal adhesion and cell adhesion molecules in LARC tissues. Among the significantly enriched KEGG gene sets in the all three cohorts, the DNA replication gene set was the most significantly involved, suggesting impairment of DNA replication by CRT.

MMR is an important and highly conserved biological process that functions to maintain genomic stability.^47^ Notably, the MMR gene set was significantly involved in all three cohorts in this study. Moreover, CRT downregulated MMR system-related genes in the three CRC cell lines examined in this study. Interestingly, 5-FU deepened 10 Gy radiation-induced downregulation of MMR system-related genes in DLD-1 and HCT-116 cells. On the other hand, 20 Gy in two fractions did not affected by 5-FU in the three CRC cell lines (figure 3D). These results should be interpreted with caution because each CRC cell line have different genetic background and CRC cell lines do not always fully reflect the genetic characteristics of LARC. Dose and fractionation regimens of radiotherapy play a crucial role in radiation-induced immunogenic cell death and efficacy of combined treatment with ICD. For example, fractionated radiotherapy (8 Gy x 3) stimulates IFN-1 signal more than single-dose radiotherapy (20 Gy x 1).^48^ Although our experimental conditions of CRT dose not match the reported regimens of CRT, MMR system-related genes were decreased by CRT. These results might be helpful in establishing guideline for combining CRT and immunotherapy.

Importantly, dysregulation of MMR system-related genes causes MSI,^49^ and the four MMR proteins (MLH1, MSH2, MSH6 and PMS2) showing loss in this study by IHC or PCR are important established biomarkers that can predict the response to ICB.^50 51^ Therefore, we also investigated changes in the MMR system-related proteins and MSI status in LARC tissues. Our results revealed that CRT induced the loss of MSH6 protein and changed the MSI status from MSS to MSI-low in LARC tissue. In samples from the same patient showing loss of MSH6 protein by IHC, the mRNA expression levels of MSH2, MSH6 and PMS2 were downregulated, and that of MLH1 was upregulated (online supplemental figure 1A), marked with red dots). This discrepancy between the results of RNA-seq and IHC may be related to differences in the post-transcriptional regulation of genes and sample status used in RNA-seq and IHC.

A higher TMB is an effective and independent predictive biomarker of responsiveness to ICB in various cancers.^15^ Our results showed that CRT significantly increased TMB in our LARC cohort, suggesting that CRT may have the potential to increase responsiveness to ICB. A recent study showed that concurrent CRT in esophageal squamous cell carcinoma (ESCC) induced immunogenomic changes, including increased immune scores, enriched immune signaling pathways and increased neutrophil proportions^52^; however, CRT significantly reduced TMB in ESCC tissues after CRT. These outcomes were somewhat different from our results because of differences in the genetic characteristics, CRT regimens and sample collection time points between the two studies. Alternatively, immune biomarker scores may also have applications as biomarkers to predict responsiveness to ICB in cancer.^16–18^ In the current study, we found that CRT not only significantly increased the cytolytic activity and the immune signature scores but also tended to increase the IFN-γ signature score in both cohorts.

Our study had some limitations. First, the RNA-seq data included only a small number of samples owing to CRT-induced necrosis of tumors. Additionally, few samples were available for IHC analysis because of weak expression and inconsistent results for MSI and MMR system-related genes. TMB analysis using the RNA-seq dataset also did not include sufficient exome sequencing. Finally, survival analysis was difficult because the time after patients enrolled was insufficient, and the experimental model of CRC cell lines was used for evaluation on the single dose of chemotherapy 5-FU (2.5 µM) and the two doses of radiation (10 or 20 Gy X-rays radiation in one or two fractions). Therefore, future studies are needed to confirm and validate our findings. Nevertheless, our findings provided strong in silico and vitro evidence of changes in bioinformatics biomarkers of the response to immunotherapy induced by CRT in patients with LARC. These results provide a molecular basis for combined treatment with radiation and immunotherapy in patients with LARC.

10.1136/jitc-2020-001610.supp6 Supplementary data

10.1136/jitc-2020-001610.supp7 Supplementary data
