# Supplementary material for: Neoadjuvant chemoradiation alters biomarkers of anticancer immunotherapy responses in locally advanced rectal cancer
Source: J Immunother Cancer. 2021 Mar 10;9(3):e001610. doi: 10.1136/jitc-2020-001610 (PMC7949478; doi:10.1136/jitc-2020-001610)
Supplement: Supplementary data [file jitc-2020-001610supp001.pdf]

Supplementary Table 1. Characteristics and postoperative pathologic outcomes of patients in our LARC cohort

Case_ID	Age	Sex	TRG Mandard _G	MSI Testing	BMI	cT stage	ypT stage	cN stage	ypN stage	Differentiation	Tumor budding	Perineural invasion (PNI)	Extranodal tumor deposit	Exon 2 K-ras mutation	BRAF mutation
1	69	M	NR	L	23.9	2	1	1	0	1	N	N	N	Y	N
2	47	M	NR	L	26.3	4	1	2	0	1	N	N	N	N	N
3	66	M	NR	L	20.9	3	3	1	0	1	N	N	N	Y	N
4	72	M	R	H	23.8	3	0	0	0	1	N	N	N	N	Y
5	67	M	NR	L	26.2	3	3	2	0	1	N	Y	N	N	N
6	72	M	R	L	19.5	3	0	1	0	1	N	N	N	Y	N
7	72	M	NR	L	22.9	4	3	2	2	1	N	N	Y	N	N
8	55	M	R	H	18.9	3	2	2	0	2	N	N	N	N	N
10	68	M	NR	L	20.3	3	3	0	0	1	Y	N	N	N	N
12	54	M	R	L	29.0	3	0	1	0	1	N	N	N	Y	N
14	76	M	NR	L	21.3	3	3	2	1	1	N	N	N	N	N

M, male; R, response to neoadjuvant chemoradiation; NR, non-response to neoadjuvant chemoradiation; H, microsatellite instability-high; L, microsatellite instability-low; MSI, microsatellite instability; BMI, body mass index; cT, clinical primary tumor; ypT, posttreatment primary tumor; cN, clinical regional lymph node; ypN, posttreatment regional lymph node; LARC, locally advanced rectal cancer; N, no; Y, yes
