# Supplementary material for: Neoadjuvant chemoradiation alters biomarkers of anticancer immunotherapy responses in locally advanced rectal cancer
Source: J Immunother Cancer. 2021 Mar 10;9(3):e001610. doi: 10.1136/jitc-2020-001610 (PMC7949478; doi:10.1136/jitc-2020-001610)
Supplement: Supplementary data [file jitc-2020-001610supp008.pdf]

Supplementary Method 1. Preoperative chemoradiation regimen of patients in our LARC cohort.

Patients underwent fluoropyrimidine-based CONCURRENT chemoradiation therapy prior to surgery. A dose of 50.4 Gy was given, which included 45 Gy in 25 fractions for the pelvis and a 5.4 Gy boost in 3 fractions for RESIDUAL primary tumor(s) over 5.5 weeks. The chemotherapy regimens are: 2 cycles of intravenous bolus of fluorouracil (400 mg/m^2 per day) and leucovorin (20 mg/m^2 per day) for 5 days in the first and fifth weeks of radiation therapy.
