# Supplementary material for: Neoadjuvant chemoradiation alters biomarkers of anticancer immunotherapy responses in locally advanced rectal cancer
Source: J Immunother Cancer. 2021 Mar 10;9(3):e001610. doi: 10.1136/jitc-2020-001610 (PMC7949478; doi:10.1136/jitc-2020-001610)
Supplement: Supplementary data [file jitc-2020-001610supp002.pdf]

Supplementary Table 2. Read lengths.

ID	Raw reads	Reads after trimming	Mapped reads
1-PRERT	18,058,563	16,733,633 (92.66)	16,364,242 (97.79)
1-POSTRT	20,301,918	18,765,530 (92.43)	18,239,808 (97.20)
2-PRERT	16,825,688	15,542,904 (92.38)	15,178,983 (97.66)
2-POSTRT	18,838,467	17,544,965 (93.13)	17,072,947 (97.31)
3-PRERT	16,907,244	15,676,022 (92.72)	15,333,695 (97.82)
3-POSTRT	20,538,117	19,021,628 (92.62)	18,565,691 (97.60)
4-PRERT	18,861,843	17,519,576 (92.88)	17,175,773 (98.04)
4-POSTRT	19,061,963	17,662,984 (92.66)	17,254,851 (97.69)
5-PRERT	17,305,382	16,216,182 (93.71)	15,913,698 (98.13)
5-POSTRT	17,803,704	16,501,411 (92.69)	16,000,822 (96.97)
6-PRERT	18,461,008	16,971,725 (91.93)	16,609,666 (97.87)
6-POSTRT	19,629,324	18,158,133 (92.51)	17,711,303 (97.54)
7-PRERT	17,973,926	16,020,523 (89.13)	15,631,032 (97.57)
7-POSTRT	18,535,525	17,099,368 (92.25)	16,661,351 (97.44)
8-PRERT	17,792,102	16,057,480 (90.25)	15,706,844 (97.82)
8-POSTRT	17,698,298	16,334,788 (92.30)	15,803,813 (96.75)
10-PRERT	17,996,452	16,730,855 (92.97)	16,390,228 (97.96)
10-POSTRT	19,363,116	17,885,076 (92.37)	17,454,124 (97.59)
12-PRERT	18,641,037	17,177,027 (92.15)	16,753,695 (97.54)
12-POSTRT	16,671,573	13,756,025 (82.51)	13,098,322 (95.22)
14-PRERT	18,552,789	16,459,995 (88.72)	15,759,179 (95.74)
14-POSTRT	21,771,211	20,579,574 (94.53)	20,220,822 (98.26)

Note: the minimum length of reads to be dropped was 38 bp.
