# Supplementary material for: Neoadjuvant chemoradiation alters biomarkers of anticancer immunotherapy responses in locally advanced rectal cancer
Source: J Immunother Cancer. 2021 Mar 10;9(3):e001610. doi: 10.1136/jitc-2020-001610 (PMC7949478; doi:10.1136/jitc-2020-001610)
Supplement: Supplementary data [file jitc-2020-001610supp003.pdf]

Supplementary Table 3. Number of variants called from each sample in our LARC cohort.

ID	No. of total variants	Common variants	Callable loci	TMB (somatic variants per Mbp)
1-PRERT	64,484	39,143	48,897,606	518.25
1-POSTRT	80,526	39,143	55,051,650	751.71
2-PRERT	63,193	31,316	47,655,625	668.90
2-POSTRT	78,075	31,316	53,864,994	868.08
3-PRERT	61,853	33,744	47,055,069	597.36
3-POSTRT	78,569	33,744	53,810,905	833.01
4-PRERT	61,386	33,781	46,184,868	597.71
4-POSTRT	69,435	33,781	50,501,224	706.00
5-PRERT	63,835	32,970	47,978,747	643.31
5-POSTRT	69,500	32,970	49,766,227	734.03
6-PRERT	66,198	35,764	50,631,976	601.08
6-POSTRT	70,490	35,764	50,129,685	692.72
7-PRERT	62,945	36,842	47,746,549	546.70
7-POSTRT	72,143	36,842	52,064,400	678.03
8-PRERT	45,503	27,090	38,551,811	477.62
8-POSTRT	64,777	27,090	47,335,812	796.16
10-PRERT	69,064	38,430	51,882,343	590.45
10-POSTRT	73,558	38,430	54,195,701	648.17
12-PRERT	56,591	25,743	42,621,344	723.77
12-POSTRT	58,303	25,743	44,070,288	738.82
14-PRERT	62,445	27,241	41,921,885	839.75
14-POSTRT	60,936	27,241	43,213,310	779.74

LARC, locally advanced rectal cancer; Mbp, million base pairs; TMB, tumor mutational burden
