# Supplementary figures and images for: Neoadjuvant chemoradiation alters biomarkers of anticancer immunotherapy responses in locally advanced rectal cancer
Source: J Immunother Cancer. 2021 Mar 10;9(3):e001610. doi: 10.1136/jitc-2020-001610 (PMC7949478; doi:10.1136/jitc-2020-001610)

Supplementary figure 2A

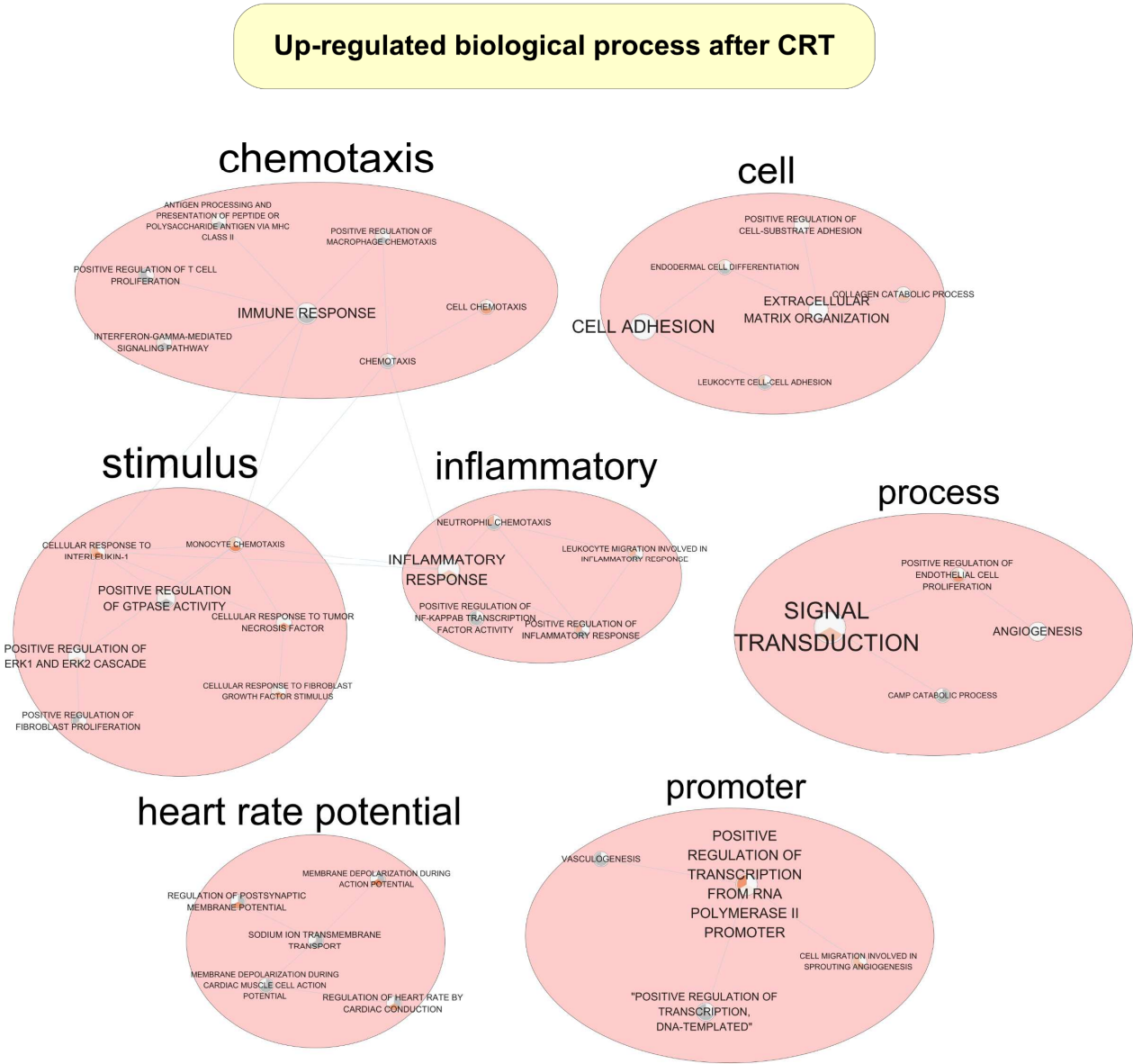

Supplementary figure 2B

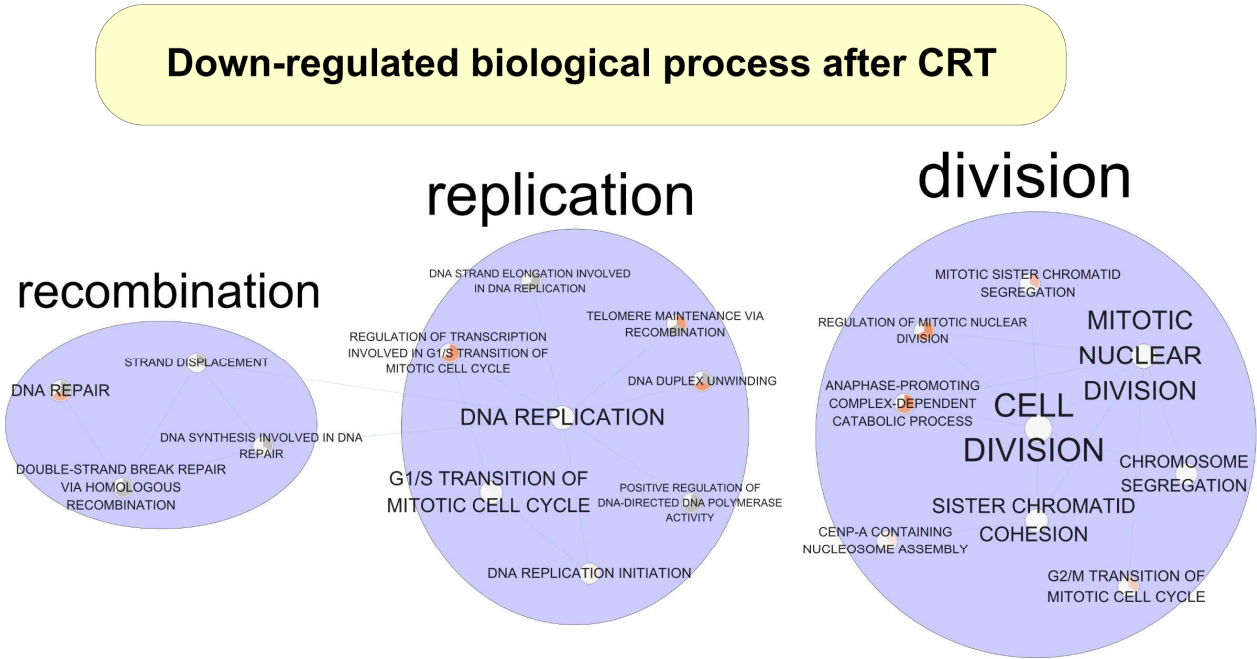

Supplementary figure 2C

RNAseq

GSE15781

GSE94104

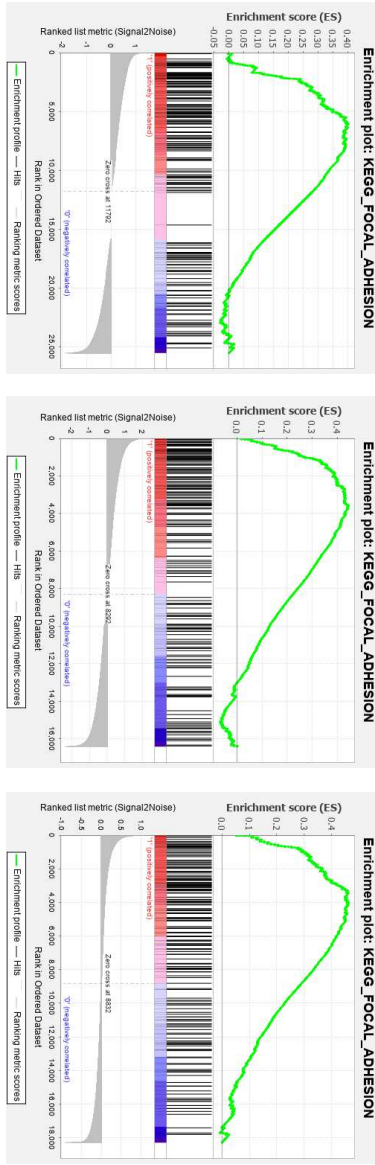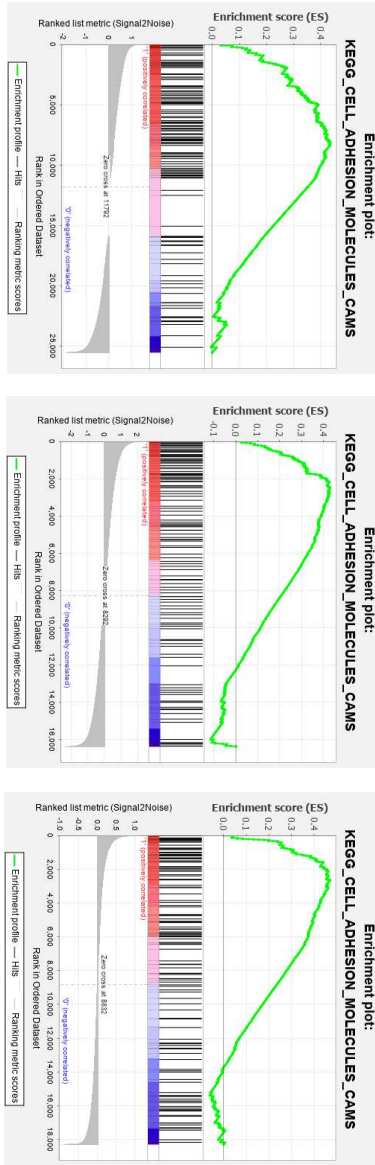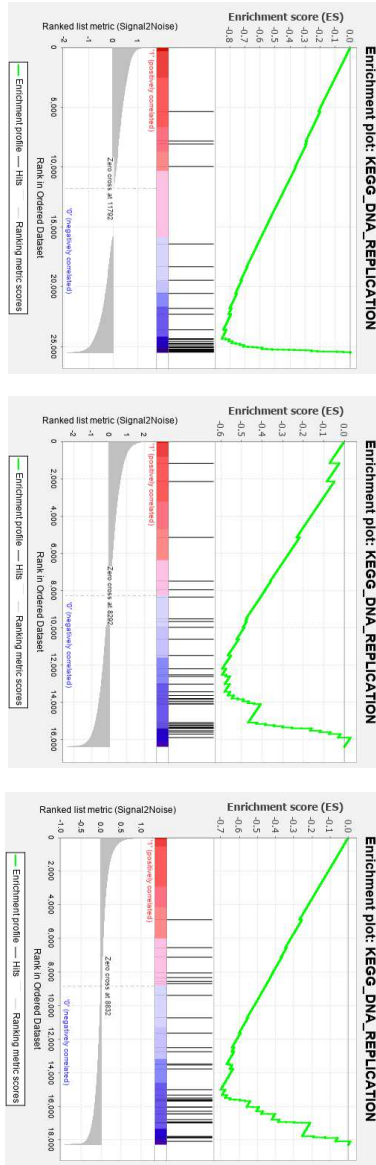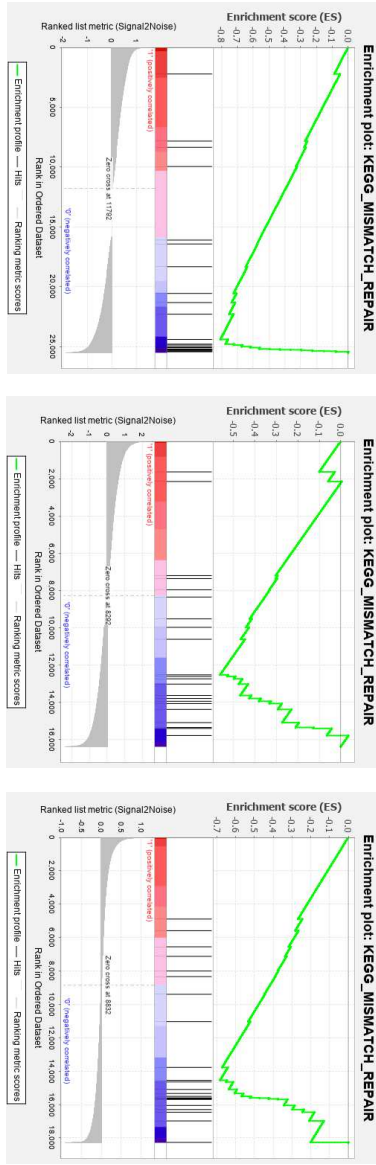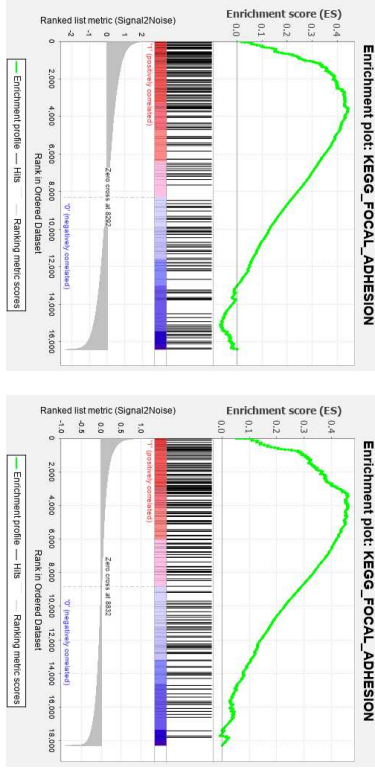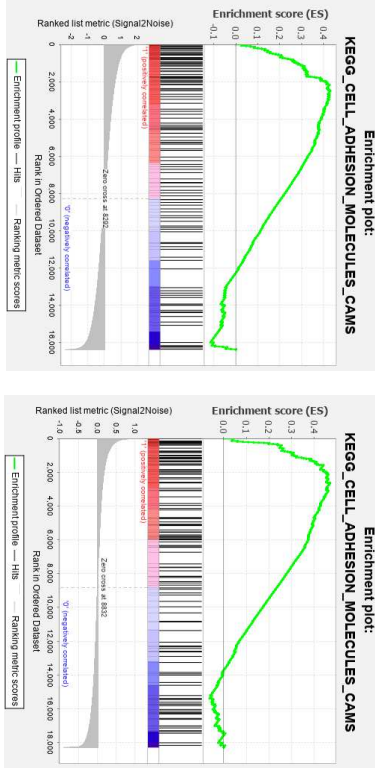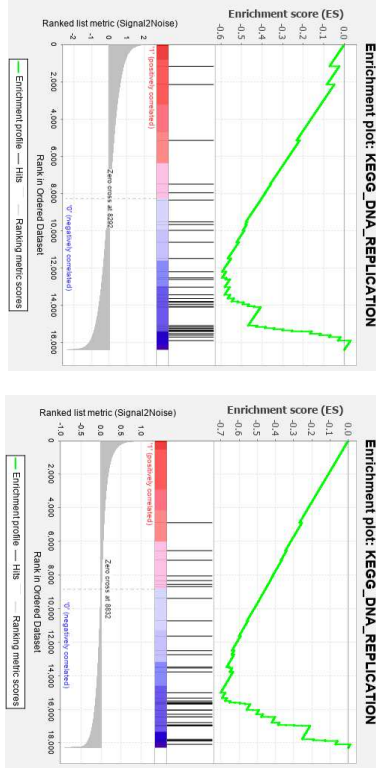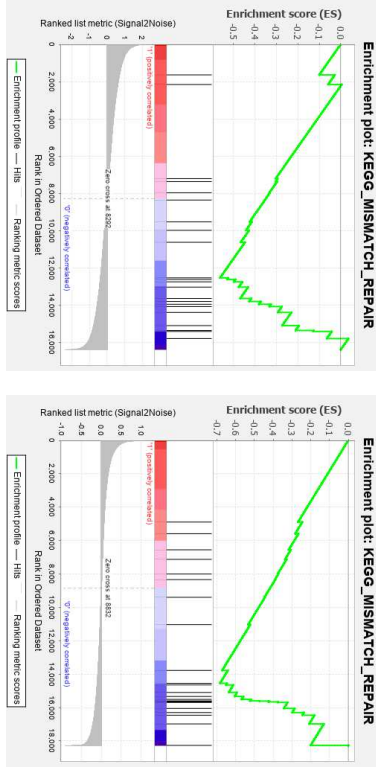

Supplement: Supplementary data [file jitc-2020-001610supp004.pdf]

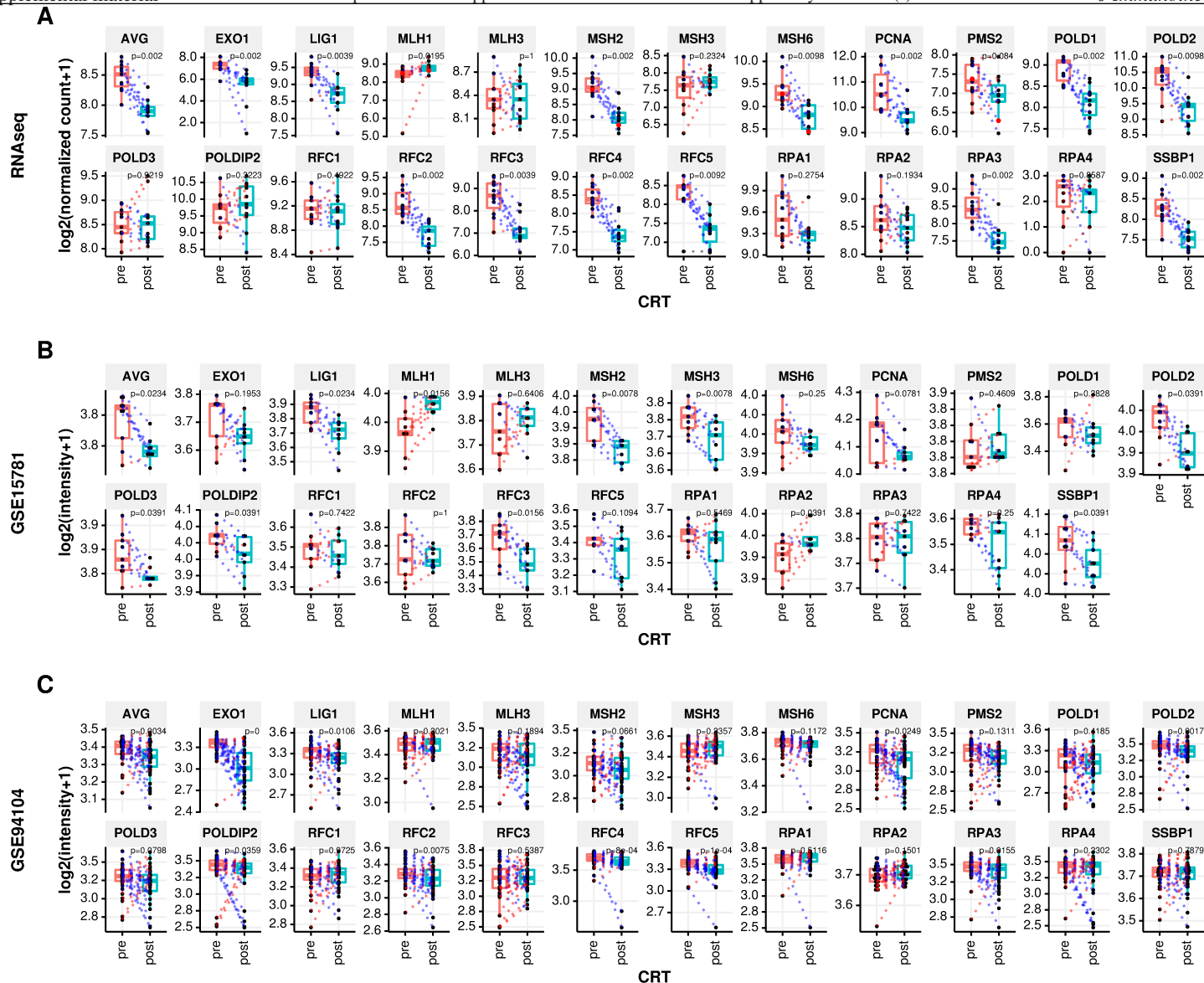

Supplement: Supplementary data [file jitc-2020-001610supp006.pdf]
