# Supplementary material for: Neoadjuvant chemoradiation alters biomarkers of anticancer immunotherapy responses in locally advanced rectal cancer
Source: J Immunother Cancer. 2021 Mar 10;9(3):e001610. doi: 10.1136/jitc-2020-001610 (PMC7949478; doi:10.1136/jitc-2020-001610)
Supplement: Supplementary data [file jitc-2020-001610supp005.pdf]

Supplementary Table 4. Significantly enriched biological processes in GSEA of the three cohorts.

Supplementary Table 4-1. Significantly enriched biological processes in GSEA of the RNA-seq cohort

Biological process	Size	ES	NES	Nom *p* value	FDR q-value	FWER *p* value
DILATED_CARDIOMYOPATHY	90	0.5125893	1.9825155	0	0.006476901	0.006
CALCIUM_SIGNALING_PATHWAY	175	0.46418902	1.9757535	0	0.003748867	0.007
HYPERTROPHIC_CARDIOMYOPATHY_HCM	83	0.50671226	1.908026	0	0.007400138	0.021
HEMATOPOIETIC_CELL_LINEAGE	86	0.4956405	1.8727208	0	0.008608614	0.032
VASCULAR_SMOOTH_MUSCLE_CONTRACTION	114	0.4644208	1.8658024	0	0.006886891	0.032
ARRHYTHMOGENIC_RIGHT_VENTRICULAR_ CARDIOMYOPATHY_ARVC	74	0.49870643	1.8555245	0	0.007351546	0.041
ECM_RECEPTOR_INTERACTION	84	0.4827511	1.8464414	0	0.007072384	0.046
ASTHMA	30	0.6003459	1.8422378	0	0.006594927	0.049
GRAFT_VERSUS_HOST_DISEASE	41	0.5428953	1.7973992	0	0.010876824	0.09
AUTOIMMUNE_THYROID_DISEASE	51	0.51586497	1.7940958	0	0.010505675	0.097
CELL_ADHESION_MOLECULES_CAMS	130	0.43658504	1.7939343	0	0.009550614	0.097
NEUROACTIVE_LIGAND_RECEPTOR_INTERACTION	270	0.3942687	1.7656447	0	0.012545356	0.14
REGULATION_OF_AUTOPHAGY	34	0.5398197	1.7385498	0.003824092	0.01590422	0.186
FOCAL_ADHESION	197	0.4028898	1.7380211	0	0.014768204	0.186
ALLOGRAFT_REJECTION	37	0.5302213	1.7213805	0.001934236	0.016476285	0.217
LONG_TERM_DEPRESSION	70	0.46136054	1.7079692	0	0.017353414	0.238
LONG_TERM_POTENTIATION	70	0.43724975	1.6331328	0.003816794	0.03305391	0.419
OLFACTORY_TRANSDUCTION	379	0.35552466	1.6294472	0	0.032004103	0.428
SPLICEOSOME	125	-0.6826143	-2.7658868	0	0	0
DNA_REPLICATION	36	-0.8515296	-2.7587698	0	0	0

CELL_CYCLE	124	-0.6641761	-2.6726406	0	0	0
RIBOSOME	84	-0.66559803	-2.5256746	0	0	0
BASE_EXCISION_REPAIR	33	-0.7684398	-2.4396727	0	0	0
PROTEASOME	43	-0.7071348	-2.4239998	0	0	0
MISMATCH_REPAIR	23	-0.80656695	-2.3662093	0	0	0
AMINOACYL_TRNA_BIOSYNTHESIS	39	-0.7060635	-2.3642282	0	0	0
HOMOLOGOUS_RECOMBINATION	26	-0.77329946	-2.327796	0	0	0
PYRIMIDINE_METABOLISM	97	-0.58644736	-2.3116546	0	0	0
RNA_POLYMERASE	29	-0.73242897	-2.261553	0	0	0
RNA_DEGRADATION	53	-0.620992	-2.1442883	0	0	0
NUCLEOTIDE_EXCISION_REPAIR	44	-0.617002	-2.063633	0	0	0
N_GLYCAN_BIOSYNTHESIS	46	-0.5494952	-1.8698767	0	0.002188098	0.032
ALANINE_ASPARTATE_AND_GLUTAMATE_METABOLISM	31	-0.5881689	-1.8669325	0	0.002169371	0.034
CYSTEINE_AND_METHIONINE_METABOLISM	34	-0.57071894	-1.8656715	0	0.002088731	0.035
PURINE_METABOLISM	155	-0.43708715	-1.8541654	0	0.002225027	0.04
OOCYTE_MEIOSIS	111	-0.45886746	-1.832368	0	0.002783426	0.052
BASAL_TRANSCRIPTION_FACTORS	35	-0.5689937	-1.8084297	0.002066116	0.003596376	0.07
SELENOAMINO_ACID_METABOLISM	25	-0.6029439	-1.8009231	0	0.003688005	0.076
PENTOSE_PHOSPHATE_PATHWAY	26	-0.57771033	-1.7341917	0.004166667	0.007883973	0.162
PROGESTERONE_MEDIATED_OOCYTE_MATURATION	85	-0.44431958	-1.6868293	0.002118644	0.012527867	0.26
GLYOXYLATE_AND_DICARBOXYLATE_METABOLISM	16	-0.6329198	-1.6816388	0.012552301	0.012731631	0.274
PEROXISOME	78	-0.43489367	-1.6427	0.00212766	0.018011903	0.377
ONE_CARBON_POOL_BY_FOLATE	17	-0.59410673	-1.6189371	0.018907564	0.02206849	0.445
STEROID_BIOSYNTHESIS	16	-0.5842019	-1.557012	0.03050109	0.035570364	0.638
HUNTINGTONS_DISEASE	160	-0.364217	-1.5333275	0	0.0424422	0.719
CITRATE_CYCLE_TCA_CYCLE	30	-0.48704192	-1.5312638	0.01629328	0.041356437	0.724

ES, enrichment score; FDR, false-discovery rate; FWER, familywise-error rate; GSEA, gene set enrichment analysis; NES, normalized

enrichment score

Supplementary Table 4-2. Significantly enriched biological processes in GSEA in the GSE15781 cohort

Biological process	Size	ES	NES	Nom *p* value	FDR q-value	FWER *p* value
ASTHMA	28	0.73588747	2.5179844	0	0	0
ALLOGRAFT_REJECTION	32	0.72345424	2.498432	0	0	0
GRAFT_VERSUS_HOST_DISEASE	34	0.68916994	2.3831823	0	0	0
LEISHMANIA_INFECTION	64	0.58616734	2.3514082	0	0	0
INTESTINAL_IMMUNE_NETWORK_FOR_IGA_PRODUCTION	42	0.6263371	2.304866	0	0	0
COMPLEMENT_AND_COAGULATION_CASCADES	63	0.577753	2.2939477	0	0	0
GLYCOSAMINOGLYCAN_BIOSYNTHESIS_CHONDROITIN_ SULFATE	19	0.7334301	2.2069645	0	0	0
AUTOIMMUNE_THYROID_DISEASE	46	0.5839816	2.1899981	0	0	0
FOCAL_ADHESION	186	0.445508	2.1271925	0	2.17E-04	0.002
ECM_RECEPTOR_INTERACTION	77	0.5060727	2.0891776	0	1.96E-04	0.002
VIRAL_MYOCARDITIS	63	0.5139724	2.016566	0	8.57E-04	0.01
CYTOKINE_CYTOKINE_RECEPTOR_INTERACTION	243	0.41357958	2.0133467	0	8.63E-04	0.011
HEMATOPOIETIC_CELL_LINEAGE	80	0.48226136	1.9927986	0	0.001312858	0.018
CELL_ADHESION_MOLECULES_CAMS	122	0.4314273	1.9497426	0	0.002034219	0.03
TYPE_I_DIABETES_MELLITUS	38	0.53817123	1.9339674	0	0.002226526	0.034
SNARE_INTERACTIONS_IN_VESICULAR_TRANSPORT	35	0.5134649	1.8601531	0	0.004540115	0.07
VASCULAR_SMOOTH_MUSCLE_CONTRACTION	103	0.43035984	1.8598087	0	0.00427305	0.07
CHEMOKINE_SIGNALING_PATHWAY	172	0.39330772	1.8331753	0	0.005704433	0.098

LEUKOCYTE_TRANSENDOTHELIAL_MIGRATION	106	0.4017337	1.7941904	0	0.008774756	0.155
SYSTEMIC_LUPUS_ERYTHEMATOSUS	106	0.390597	1.7322614	0	0.013767486	0.249
NOD_LIKE_RECEPTOR_SIGNALING_PATHWAY	62	0.43366337	1.7293886	0	0.013427097	0.253
PRION_DISEASES	35	0.4806068	1.6911408	0.003875969	0.018698048	0.351
GLYCOSPHINGOLIPID_BIOSYNTHESIS_GANGLIO_SERIES	15	0.60667235	1.6904539	0.0056926	0.018050753	0.352
MAPK_SIGNALING_PATHWAY	253	0.3397241	1.6634429	0	0.022204485	0.435
REGULATION_OF_ACTIN_CYTOSKELETON	195	0.33810678	1.6221337	0	0.03071796	0.564
HYPERTROPHIC_CARDIOMYOPATHY_HCM	79	0.38849574	1.6161528	0.003546099	0.031022798	0.578
EPITHELIAL_CELL_SIGNALING_IN_HELICOBACTER_PYLORI_INFECTION	66	0.39971268	1.5803355	0.01934236	0.04046661	0.697
NATURAL_KILLER_CELL_MEDIATED_CYTOTOXICITY	123	0.3486758	1.5708547	0	0.042244826	0.726
MELANOMA	69	0.38169178	1.5702761	0.009578544	0.041019298	0.728
NEUROTROPHIN_SIGNALING_PATHWAY	120	0.35502195	1.57002	0.003649635	0.03977624	0.729
RIG_I_LIKE_RECEPTOR_SIGNALING_PATHWAY	64	0.39188373	1.5609279	0.013916501	0.041359786	0.756
DILATED_CARDIOMYOPATHY	85	0.36926553	1.5565197	0.007648184	0.041745313	0.774
PATHWAYS_IN_CANCER	316	0.30397028	1.5490415	0	0.042838704	0.788
ANTIGEN_PROCESSING_AND_PRESENTATION	76	0.37691656	1.5369248	0.007462686	0.046710495	0.825
LYSOSOME	115	0.34756178	1.5245861	0.007490637	0.049622618	0.847
TOLL_LIKE_RECEPTOR_SIGNALING_PATHWAY	100	0.35285708	1.5229201	0.001901141	0.048963655	0.848
CITRATE_CYCLE_TCA_CYCLE	30	-0.62892634	-2.1918447	0	0	0
AMINOACYL_TRNA_BIOSYNTHESIS	32	-0.60830474	-2.1323717	0	0	0
DNA_REPLICATION	33	-0.5951376	-2.1039531	0	0	0
BASE_EXCISION_REPAIR	33	-0.58671963	-2.035338	0	0.001006253	0.004
PYRIMIDINE_METABOLISM	90	-0.46414363	-2.0068944	0	0.001411438	0.007
SPLICEOSOME	113	-0.44299504	-1.9966822	0	0.00172984	0.01
DRUG_METABOLISM_OTHER_ENZYMES	39	-0.52041477	-1.8975196	0	0.006189106	0.04
STEROID_BIOSYNTHESIS	16	-0.6416227	-1.8479576	0.002132196	0.010840432	0.079

PEROXISOME	75	-0.43664226	-1.8302426	0	0.012082151	0.098
RNA_POLYMERASE	26	-0.550258	-1.7956698	0.011857707	0.015288933	0.13
VALINE_LEUCINE_AND_ISOLEUCINE_DEGRADATION	42	-0.47456235	-1.7846826	0.004140787	0.015903868	0.149
MISMATCH_REPAIR	22	-0.5634375	-1.7611866	0.002079002	0.018849427	0.191
TERPENOID_BACKBONE_BIOSYNTHESIS	15	-0.6244792	-1.7600522	0.006147541	0.017642075	0.192
ALANINE_ASPARTATE_AND_GLUTAMATE_METABOLISM	31	-0.4917142	-1.7519854	0.004338395	0.017693076	0.204
CELL_CYCLE	122	-0.3796363	-1.74143	0	0.018191693	0.224
SELENOAMINO_ACID_METABOLISM	21	-0.53938395	-1.7279283	0.010416667	0.018887721	0.246
RIBOSOME	75	-0.40516287	-1.7208233	0	0.018710503	0.259
PARKINSONS_DISEASE	97	-0.39299116	-1.7123795	0	0.019501364	0.278
GLYCOSPHINGOLIPID_BIOSYNTHESIS_LACTO_AND_NEOLACTO_SERIES	26	-0.5284643	-1.7038212	0.008639309	0.019893743	0.295
GLUTATHIONE_METABOLISM	44	-0.44670752	-1.6992595	0	0.019898029	0.312
HUNTINGTONS_DISEASE	150	-0.35769758	-1.6822829	0	0.021907598	0.348
PROXIMAL_TUBULE_BICARBONATE_RECLAMATION	23	-0.5226012	-1.6757207	0.015151516	0.02179761	0.359
PROPANOATE_METABOLISM	30	-0.46956128	-1.6182915	0.00631579	0.034719527	0.525
THYROID_CANCER	28	-0.4587294	-1.5823514	0.022177419	0.045327876	0.643

ES, enrichment score; FDR, false-discovery rate; FWER, familywise-error rate; GSEA, gene set enrichment analysis; NES, normalized enrichment score

Supplementary Table 4-3. Significantly enriched biological processes in GSEA in the GSE94104 cohort

Biological process	Size	ES	NES	Nom *p* value	FDR q-value	FWER *p* value

DILATED_CARDIOMYOPATHY	86	0.5738871	2.09093	0	9.49E-04	0.001
MELANOMA	67	0.58874094	2.0711582	0	9.36E-04	0.002
HYPERTROPHIC_CARDIOMYOPATHY_HCM	79	0.56813705	2.0289567	0	6.24E-04	0.002
CALCIUM_SIGNALING_PATHWAY	162	0.49308497	1.9452127	0	0.003931471	0.016
ECM_RECEPTOR_INTERACTION	82	0.53636074	1.9236966	0	0.004036177	0.021
FOCAL_ADHESION	189	0.46129414	1.8581474	0	0.008099408	0.051
ARRHYTHMOGENIC_RIGHT_VENTRICULAR_ CARDIOMYOPATHY_ARVC	71	0.5191158	1.8241311	0.00170068	0.010827938	0.077
CELL_ADHESION_MOLECULES_CAMS	131	0.46934408	1.8116379	0	0.011433065	0.092
VIRAL_MYOCARDITIS	63	0.5030668	1.7179166	0.001814882	0.03081232	0.266
DNA_REPLICATION	36	-0.7007715	-2.3010209	0	0	0
CELL_CYCLE	122	-0.57120323	-2.295487	0	0	0
MISMATCH_REPAIR	23	-0.6837358	-2.020403	0	7.03E-04	0.002
PEROXISOME	75	-0.5047265	-1.8933221	0	0.005765674	0.021
ASCORBATE_AND_ALDARATE_METABOLISM	20	-0.6515238	-1.8501037	0	0.008128142	0.037
BASE_EXCISION_REPAIR	33	-0.57392	-1.8270599	0.002352941	0.008980554	0.047
MATURITY_ONSET_DIABETES_OF_THE_YOUNG	22	-0.62876385	-1.8215218	0.002173913	0.008178297	0.05
RIBOSOME	81	-0.46520224	-1.7553846	0	0.016389811	0.112
HOMOLOGOUS_RECOMBINATION	28	-0.57828945	-1.7548293	0.002159827	0.014813413	0.114
PENTOSE_AND_GLUCURONATE_INTERCONVERSIONS	23	-0.59495944	-1.7142693	0.002257336	0.0222204	0.181
PORPHYRIN_AND_CHLOROPHYLL_METABOLISM	34	-0.5163599	-1.672384	0.011185682	0.03282529	0.276
RIBOFLAVIN_METABOLISM	16	-0.6053013	-1.637684	0.018907564	0.04302932	0.371
FRUCTOSE_AND_MANNOSE_METABOLISM	34	-0.5114403	-1.6306827	0.013157895	0.042943906	0.394
DRUG_METABOLISM_OTHER_ENZYMES	41	-0.48197663	-1.6222355	0.004237288	0.04386709	0.425

ES, enrichment score; FDR, false-discovery rate; FWER, familywise-error rate; GSEA, gene set enrichment analysis; NES, normalized enrichment score
