# Supplementary material for: Neoadjuvant chemoradiation alters biomarkers of anticancer immunotherapy responses in locally advanced rectal cancer
Source: J Immunother Cancer. 2021 Mar 10;9(3):e001610. doi: 10.1136/jitc-2020-001610 (PMC7949478; doi:10.1136/jitc-2020-001610)
Supplement: Supplementary data [file jitc-2020-001610supp007.pdf]

Supplemental Figure Legends

Supplementary Figure 1. Changes in mRNA expression profiles of mismatch repair (MMR) system-related genes in locally advanced rectal cancer (LARC) tissues before and after chemoradiation therapy (CRT). A, Expression levels (log_2[normalized count+1]) of 23 MMR system-related genes in the RNA-seq cohort of LARC tissues before and after CRT. B, Expression levels (log_2[normalized count+1]) of 23 MMR system-related genes in the GSE15781 cohort of LARC tissues before and after CRT. C, Expression levels (log_2[normalized count+1]) of 23 MMR system-related genes in the GSE94104 cohort of LARC tissues before and after CRT. *P values were calculated by Wilcoxon signed-rank tests. Pre, LARC tissues before CRT; Post, LARC tissues after CRT.

Supplementary Figure 2. Functional enrichment analysis of differentially expressed genes (DEGs) in locally advanced rectal cancer (LARC) tissues before and after CRT. A, Significantly enriched biological processes in LARC tissues after CRT compared with that before CRT. B, Significantly enriched biological processes in LARC tissues before CRT compared with that after CRT. C, Gene set enrichment analysis (GSEA) plots (score curves) of the three cohorts. Representative GSEA of DEGs in tumor samples before and after CRT in the RNA-seq, GSE15781, and GSE94104 datasets.
